# The voltage-dependence of MscL has dipolar and dielectric contributions and is governed by local intramembrane electric field

**DOI:** 10.1038/s41598-018-31945-x

**Published:** 2018-09-11

**Authors:** Joseph S. Najem, Ian Rowe, Andriy Anishkin, Donald J. Leo, Sergei Sukharev

**Affiliations:** 1 Joint Institute for Biological Sciences, Oak Ridge National Laboratory, Oak Ridge, Tennessee 37830 United States; 2 Department of Mechanical, Aerospace, and Biomedical Engineering, University of Tennessee, Knoxville, Tennessee 37916 United States; 3 Department of Biology, University of Maryland, College Park, MD 20817 United States; 4 College of Engineering, University of Georgia, Athens, Georgia 30605 United States

## Abstract

Channels without canonical voltage sensors can be modulated by voltage acting on other domains. Here we show that besides protein dipoles, pore hydration can be affected by electric fields. In patches, both WT MscL and its V23T mutant show a decrease in the tension midpoint with hyperpolarization. The mutant exhibits a stronger parabolic dependence of transition energy on voltage, highly consistent with the favourable dielectric contribution from water filling the expanding pore. Purified V23T MscL in DPhPC droplet interface bilayers shows a similar voltage dependence. When reconstituted in an asymmetric DOPhPC/DPhPC bilayer carrying a permanent bias of ~130 mV due to a dipole potential difference between the interfaces, the channel behaved as if the local intramembrane electric field sets the tension threshold for gating rather than just the externally applied voltage. The data emphasize the roles of polarized water in the pore and interfacial lipid dipoles in channel gating thermodynamics.

## Introduction

Transmembrane voltage is a critical parameter for most ion channels because (i) it provides the driving force for ions and (ii) it is a powerful factor in triggering and modulating channel gating. Channels carrying canonical voltage sensor domains with multiple charges change their open probability steeply with voltage^1,2^. However, channels that are not considered to be voltage-gated and lack specialized voltage sensors can also be modulated by voltage acting on other domains. The reason is that a typical channel residing in the polarized membrane represents a non-uniform protein insert characterized by local parameters of dielectric permeability and electric charge whose distributions may change as the channel transitions between the states. Here we describe a strong modulation of the bacterial mechanosensitive channel MscL and especially its mild gain-of-function mutant (V23T)^3^, which is apparently exerted through a non-specific dielectric mechanism by water present in the pore, with some contribution from the dipole moment of the protein itself. Previously, this mutation was suggested to produce excessive solvation and partial expansion of the normally vapour-locked MscL pore^4^.

MscL is ubiquitously found in bacteria and archaea and functions as a tension-activated osmolyte release valve in the cytoplasmic membrane. The channel opens at tensions approaching the lytic limit (10–14 mN/m)^5,6^ and thus acts as an emergency valve that rescues bacteria from abrupt osmotic shock^7^. Under strong osmotic shocks membrane permeability increases by orders of magnitude for only tens of milliseconds^8^. Under resting conditions, however, the cytoplasmic membrane acting as an energy-coupling insulator usually remains uncompromised^9^. It is important that the fully energized cytoplasmic membrane maintains a high (150–220 mV) membrane potential^10^, so the question is whether MscL’s high activation tension threshold is influenced by voltage in any way, and if so, how does the system avoid leakage.

On the other hand, with its relatively simple structural design^11,12^, MscL has been considered a promising precursor for the development of controllable molecular valves for drug delivery^13^. It has also been proposed as a possible component of autonomic sensory devices and ‘smart’ responsive materials^14–16^. The principles and mechanisms of electric field effects on the conformational equilibrium in MscL should be equally well understood from the position of its *in-vivo* function as a bacterial ‘safety valve’, as well as from the point of view of bio-inspired material engineering.

The high activation threshold of WT *E*. *coli* MscL precluded its studies in artificial solvent-containing membranes (BLM or droplet interface bilayers (DIBs)^17^), and for this reason the V23T mutant, which is activated at 6–8 mN/m, was chosen for reconstitutions^15^. The previously observed higher probability of opening events in the course of cyclic mechanical stimulation of the DIB-reconstituted V23T MscL at membrane voltages above 80 mV^15^ motivated us to look more carefully at the voltage dependence of WT and V23T MscL. Here, we present patch-clamp measurements obtained under linear pressure ramp stimulation at different voltages and evaluate voltage contributions to the opening transition energy of these two channel variants. The data reveal that both versions of MscL have dipole (linear with voltage) and dielectric/capacitive (quadratic with voltage) components that contribute to the transition energy. In contrast to WT, which exhibits a larger dipole and a smaller dielectric contribution, V23T shows a dominating dielectric effect likely related to the more pronounced hydration of the mutant pore^3,4^. The increasing aqueous volume of the pore and the flattening of the protein complex impart the capacitive/dielectric energy contribution that stabilizes the expanded and subconductive states of the mutant channel. To further explore the nature of the electric field effect that influences the open probability, we have compared the V23T MscL behaviour in electrically symmetric and asymmetric DIBs where a large intrinsic electric bias was created by using lipids with different interfacial dipole potentials. The data obtained in DIBs confirm that V23T MscL senses the local intramembrane electric field in the membrane core between the interfacial layers, and thus the voltage imposed by external electrodes is sensed not directly, but rather in combination with the potential imparted by the lipids.

## Results

### MscL exhibits dipole and dielectric components of voltage dependence in patch-clamp experiments

**Figure 1:**
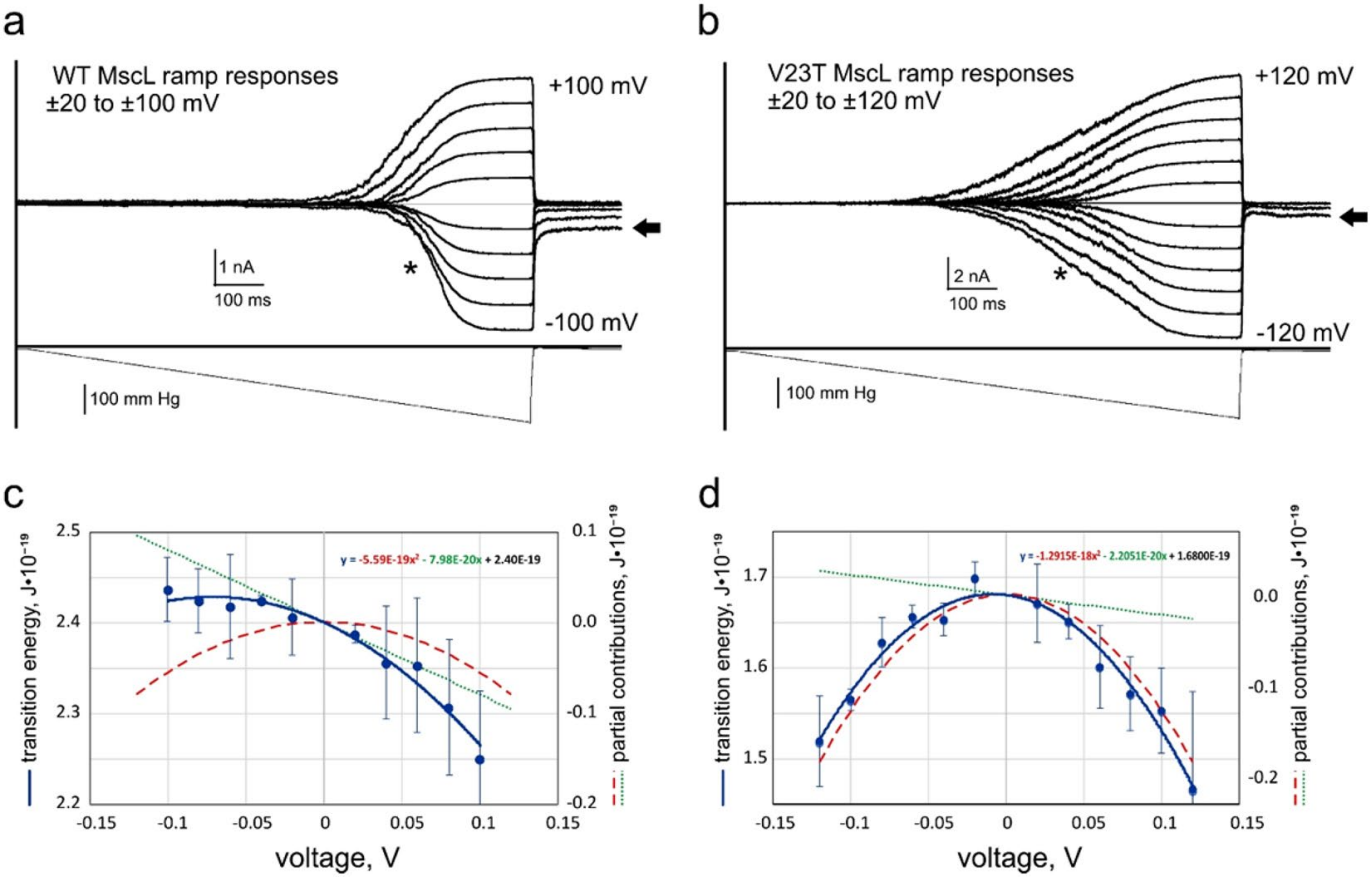
Voltage sensitivity of MscL. Current responses of WT MscL and V23T MscL populations to pressure ramps at different pipette voltages (imposed in 20 mV increments). Experiments were conducted in inside-out patches excised from giant bacterial spheroplasts expressing corresponding channels. Pressure midpoints for WT MscL (**a**) are higher at negative pipette voltages (asterisks), whereas V23T MscL (**b**) shows shallower and more symmetric responses and a more substantial shift to the left of the pressure midpoint with voltage. Both channels show ‘lingering’ conductive states at high negative voltages after stimulating pressure is released (black arrows). Plots of the closed-to-open transition energy extracted from activation curves as a function of pipette voltage (**c**,**d**). Parabolic fits predict different contributions from the capacitive (quadratic) and dipole (linear) components of the energy dependence on membrane potential, V (represented by right axes on panels c and d). This behaviour was reproduced in six independent patches for WT MscL and nine patches for V23T.

Figure 1 depicts activation curves of WT and V23T MscL populations recorded using the standard patch-clamp technique in inside-out patches excised from giant spheroplasts (~30–50 channels/patch). The mechanical stimulus is a linear ramp of pressure difference between the pipette and the bulk from zero to saturating level (~250 mm Hg), repeated at different maintained voltages. We should explain that this pressure gradient produces tension in the curved patch membrane according to the law of Laplace. This tension is the primary stimulus driving channel opening. One can see that in wild-type MscL (Fig. 1a) the midpoint for population activation does not change significantly at negative pipette voltages (asterisk) but shifts to the left with positive voltage. For V23T MscL, in contrast, the midpoint shifts considerably to the left under both voltages. The clear difference is in the slope of activation curves. For WT MscL, the curves are steeper, which correlates with higher cooperativity of gating (predominant all-or-nothing transition between the closed and fully open states), whereas the lower slope of V23T curves is ascribed to high occupancy of subconductive states at low tensions and pre-expanded closed state (i.e. smaller expansion area resulting in shallower dependence on tension)^3^. The relationships between population current are presented in Methods. We note that the tension midpoints at low voltages for WT and V23T MscL reside at 12 and 8.3 mN/m, respectively, based on previous experimental studies^3,6^. Assuming that the patch geometry (curvature) in all trials at different voltages within any particular experiment remains the same, reduction of midpoint tension γ_0.5_ can be interpreted as a reduction of the effective energy for the closed-to-open transition. We observed an unusual common feature: both channels show ‘lingering’ low-conductance states at high negative voltages after stimulating pressure is released (black arrows).

To interpret the shifts in activation curves, we assume equilibrium conditions at any tension and use the Boltzmann-type relationship for the closed and open state probabilities (*P*_*o*_ and *P*_*c*_).

1 $$\frac{{P}_{o}}{{P}_{c}}={e}^{-(Eo-\gamma {\rm{\Delta }}A)/kT}.$$

*E*_*o*_ is the free energy gap between the closed and open states in the absence of tension, γ is membrane tension, *ΔA* is the protein area change in the plane of the membrane, *k* is the Boltzmann constant, and *T* is temperature. From this relationship, one can see that at the midpoint (γ_0.5_) tension, where *P*_*o*_ is equal to *P*_*c*_, the exponential term is zero, which sets the transition energy in the absence of tension *E*_0_ = *γ*_*0*.5*_ΔA. The fact that γ _0.5_ decreases with voltage indicates that there must be voltage-dependent terms in the transition energy. Knowing midpoint tensions at low voltages and transition-associated area changes, we calculated the transition energy at each voltage. In our previous studies^3,6^, the *E*_*o*_ and Δ*A* parameters were estimated as 58 kT and 20 nm^2^ for WT MscL and *E*_*o*_ = 41 kT and *ΔA* = 18 nm^2^ for V23T MscL, respectively. As seen from Fig. 1c, the energy for WT MscL slightly increases at negative pipette voltage and decreases at positive pipette voltages. For V23T (Fig. 1d), the voltage-dependent term is always negative at both voltages (more pronounced at positive potential) and the *E*_*o*_(V) dependence can be fitted with a parabola with a dominating negative quadratic term. The parabolic fit of the WT dependence indicates a larger linear term (Fig. 1c). The linear and quadratic terms can be physically interpreted as the dipolar (*E*_*d*_) and dielectric (capacitive, *E*_*c*_) contributions of the electric polarization of the system and thus the transition energy in the absence of tension can be written as.

$${E}_{o}={E}_{i}+{E}_{d}({\rm{V}})+{E}_{c}({{\rm{V}}}^{2})$$

where *E*_*i*_ is the voltage-independent term estimated at low voltages. The open probability, therefore can be written in the form:

2 $${P}_{o}=\frac{1}{1+{e}^{\frac{{\rm{\Delta }}{E}_{i}-\gamma {\rm{\Delta }}A+{K}_{d}V+{K}_{c}{V}^{2}}{kT}}}$$

Conceptually, the constant *K*_*d*_ can be interpreted as an effective charge crossing the voltage *V*, while *K*_*c*_ as an effective capacitance that, being charged, gains energy proportional to *V*^2^. However, given that the effective dipole of the channel MscL changes during the gating transition and crosses a varying fraction of the transmembrane voltage, and also considering that the capacitance of the channel/lipid/water system is defined by heterogeneous parts with a changing geometry, interpretation of the coefficients as “fixed” effective parameters might be an oversimplification. The combination of parameters contributing to these changing coefficients is provided in the Supplement for our cylindrical multilayer model system. The shape of parabolic fitting curves of the total transition energy and fitting parameters indicate that the capacitive (dielectric) term described by the quadratic dependence on voltage dominates in V23T MscL and the dipolar (linear) term in the mutant is diminished compared to WT. Estimations based on the parabolic fits (Fig. 1c,d) show that at −100 mV pipette potential the unfavourable (opposing opening) dipole contributions constitute 1.93kT and 0.53 kT and the favourable capacitive contributions are −1.35kT and −3.11 kT for WT and V23T MscL, respectively. Given that the V23T substitution does not alter the net charge in the pentameric protein but changes the character of pore lining, these disparate electrostatic contributions raise the following questions: what is the nature of the dipole that dominates the WT MscL voltage response and how does the neutral V23T mutation diminish this dipolar contribution? Can the increased hydration of the V23T pore account for the large dielectric contribution in the mutant and where does the localized electric field exert its action in the system? Does the channel feel mostly the applied voltage between the bulk solutions or is it sensitive to the potential in a narrower spatial region where the electrostatic contribution from the membrane interfacial layers is present?

### V23T MscL exhibits voltage sensitivity dominated by the dielectric effect in droplet interface bilayers

We explored voltage-sensitivity of V23T MscL in DIBs, which allow us to form membranes with symmetric or asymmetric monolayer lipid compositions characterized with different interfacial dipole potentials^14^. Varying the local electric field inside, between the membrane’s boundary layers, would enable us to address the question related to the region where the electric field acts. In the first series of experiments we reconstituted V23T MscL into symmetric DIBs formed of DPhPC and partially reproduced the channel’s voltage dependence observed in patch-clamp experiments presented above. As previously^15^, mechanical stimulation was delivered by keeping one droplet stationary while periodically oscillating the second droplet in the axial direction with an amplitude of ±100 μm and frequency of 0.2 Hz. The most effective stimulation cycles had a shorter compression phase and longer relaxation phase characterized with the duty cycle of 75% (the fraction of cycle time taken by the relaxation phase)^16^.

**Figure 2:**
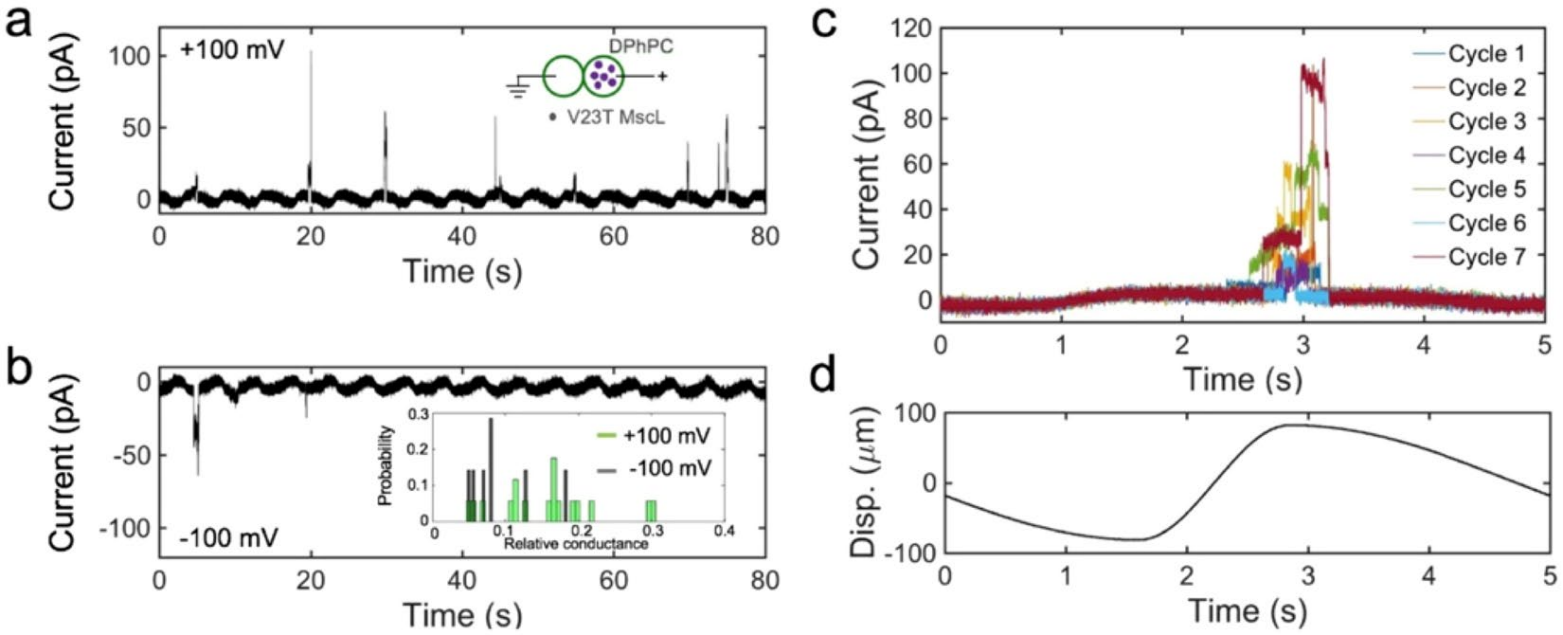
V23T MscL currents recorded in DIBs in the 0.75 duty cycle oscillating regime at different voltages. (**a**,**b**) Typical traces recorded at +100mV or −100 mV applied to the ‘cis’ compartment relative to the grounded ‘trans’ compartment. The distribution of observed conductances is shown in the inset in panel (**b**). (**c**) Seven segments of the continuous trace were recorded, each corresponding to one stimulation cycle (**d**) are overlaid to illustrate the position of opening events. As seen from panel (**c**), the channels open stochastically but tend to cluster around the 3 s time point, which is near the peak of compression where tension is highest in the membrane. In this particular trace, openings were observed in 21 out of 100 cycles.

**Figure 3:**
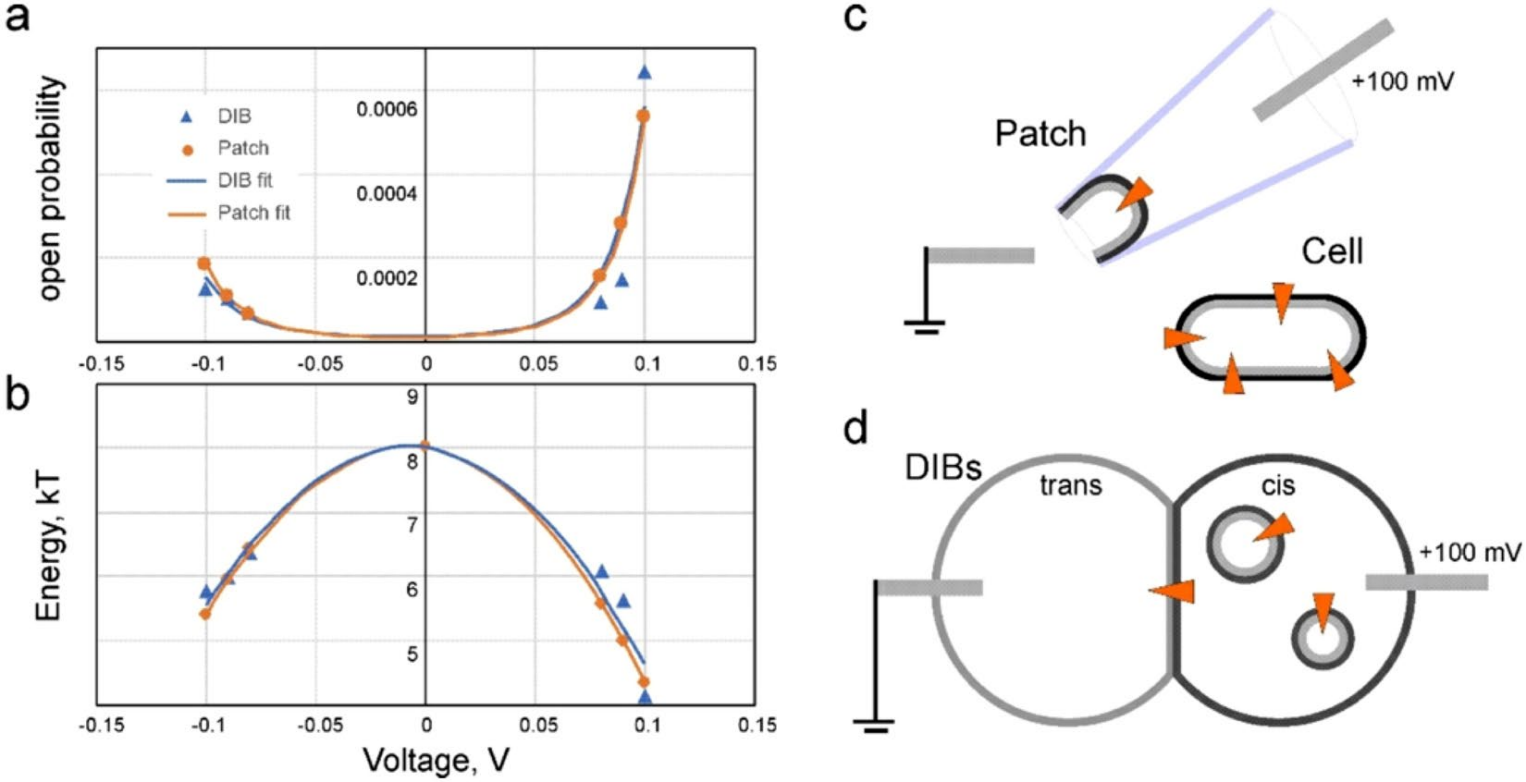
Voltage dependences of open probability (**a**) and the closed-to-open transition energy (**b**) for V23T MscL recorded in DIBs (blue symbols) and in patch-clamp experiments (orange symbols). The patch data (orange) are simulated for the membrane tension at which the DIB data are collected. The fitting of open probability was done using Eq. 2. Based on the asymmetry of voltage response (**a**,**b**) we can deduce the orientation of the MscL channel incorporated in DIBs (**c**,**d**). In patches excised from the cytoplasmic membrane (**c**) the channel remains in its native orientation with its cytoplasmic side facing the bath. The directionality of MscL incorporation into DIBs is as presented in panel (**d**) and achieved through unilateral introduction of proteoliposomes into the ‘cis’ compartment.

Figure 2 shows typical traces that were continuously recorded at +100 mV (Fig. 2a) and −100 mV (Fig. 2b) applied to the ‘cis’ compartment, i.e. droplet with V23T MscL-containing liposomes (see also Fig. 3d). The probability of seeing a channel opening event was higher at positive voltage (0.55 per cycle) than at negative voltage (0.28 per cycle). We should note that in DIBs V23T MscL opens to a variety of subconductive states, whose conductance on average was higher at +100 mV (Fig. 2b, inset). In terms of timing, opening events were well clustered near the pinnacle of compression phase, which lasts approximately 1 s (out of the entire 5 s cycle). An overlay of DIB current responses to seven selected cycles displaying openings is shown in Fig. 2c. Displacement in the positive direction (Fig. 2d) signifies that the droplets are moved closer to each other, where they get distorted from the initial near-spherical shape and generate tension. As seen from the overlaid traces, V23T MscL activates to various conductive levels and its dwell time in the open state (at +100 mV) stochastically varies between 100 and 500 ms. Although the period of time when super-threshold tension is acting on the channel is ~20% of the entire cycle duration (5 s), the cycles are identical and we presume that the open probability is proportional to the fraction of cycles in which openings were observed times the average channel conductance and open dwell time observed at that specific voltage.

Similar to patch-clamp data, increased voltage across DIB, negative or positive, increases the probability of seeing the opening event per cycle. V23T MscL, which is prone to opening to subconductive states^3^, also exhibits higher conductive states at higher voltages. These two factors, along with the dwell time in the open state, were taken into account in calculations of open probability (*P*_*o*_). Since we never observed full openings of multiple channels in DIBs, we also assumed for simplicity that we deal with a single channel incorporated into the membrane. Increase of the presumed channel population to *n* will scale down *P*_*o*_ but will not change the shape of voltage dependence.

Figure 3a depicts *P*_*o*_ as a function of voltage measured for DIBs and data from patch-clamp experiments extrapolated to the same tension. To compare two sets of data, we calculated the expected *P*_*o*_ for V23T MscL in patches under applied voltage (−100 mV to 100 mV) using the intrinsic energy cost of opening (*E*_*o*_ = 41 kT), the expansion area (*ΔA* = 18 nm^2^)^3^ and the capacitive (313.9 kT/V^2^) and dipole (5.36 kT/V) voltage sensitivities of MscL derived from the above patch-clamp measurements. With these parameters, the tension that would produce the same *P*_*o*_ in patches as observed in the DIBs system during the 0.5 s interval covering the maximum of compression phase (Fig. 2c) is estimated to be 7.34 mN/m. The estimated *P*_*o*_ for MscL in patches and *P*_*o*_ observed in DIBs (Fig. 3a) show good agreement over the whole range of applied voltages. The data were fitted with Eq. 2 and produced essentially coinciding lines despite the experimental scatter in the DIBs dataset. We have also re-calculated transition energies as functions of voltage from open probabilities obtained in DIBs and found good correspondence between the DIBs and patch-clamp data (Fig. 3b). Parabolic fits produced 288.4 kT/V^2^ for the dielectric and 4.50 kT/V for the dipole contributions to voltage sensitivity.

The similarity between asymmetric voltage sensitivities of V23T MscL in patch-clamp and DIBs systems strongly suggests that channels added in in the form of liposomes into the cis compartment insert into the interfacial bilayer directionally as depicted in Fig. 3c,d. In the excised patch configuration, the channel points with its cytoplasmic side to the bath. Liposome incorporation into the DIB’s cis compartment produces the same orientation as if the cis compartment represents the periplasmic side of the channel or the inner volume of the pipette.

### Interpretation of the dipolar and dielectric effects on MscL gating

Herein, we provide estimations of the presumed dipolar and dielectric energetic contributions to the voltage dependence of WT and V23T MscL based on molecular dimensions deduced from the closed, intermediate, and open-state models. Full details of this analysis can be found in Supplementary Information.

The general form of the energy *E*_*d*_ of a dipole $\overrightarrow{p}$ in a uniform electrostatic field $\overrightarrow{E}$ is:

3 $${E}_{d}=-\,\overrightarrow{p}\cdot \overrightarrow{E}.$$

However, the external electrostatic field around the MscL channel is non-uniform due to the different geometries and dielectric properties of the pore, the protein, and the annular lipids subjected to distortion. That said, a more convenient approximation would be to consider the dipole as a system of two-point charges (*a* and *b*) at a distance *d*, where each of them is subjected to the external field according to the electrical potential in each of the locations (*V*_*a*_ and *V*_*b*_):

4 $${E}_{d}=\frac{p}{d}({V}_{a}-{V}_{b}).$$

**Figure 4:**
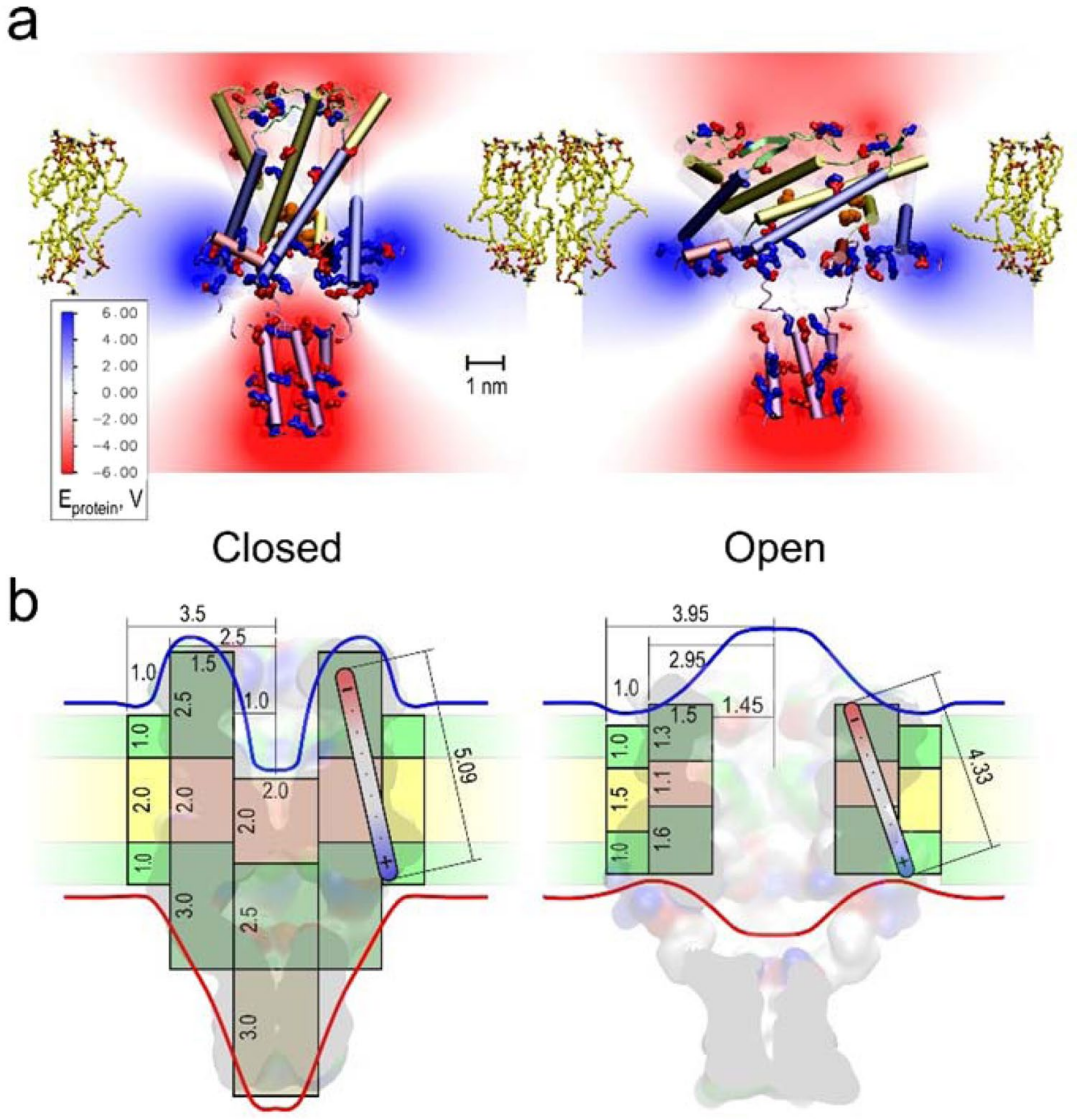
Estimations of WT MscL changes in dipole and capacitive energies as a result of channel opening. Distributions of electric potential around the MscL complex in the closed (**a**) and open (**b**) conformations. The density of red and blue colour reflects the calculated electrostatic potential of the protein, negative to positive respectively. The opening transition changes the total dipole moment of the MscL complex from 2675 D (8.92∙10^−27^ C∙m) in the closed state to 2175 D (7.25∙10^−27^ C∙m) in the open state. Simplified cylindrical representations of the closed (**c**) and open (**d**) conformations of WT MscL superimposed with the molecular models of the closed and open states^38^. Grey and pink regions represent high-dielectric (polar) and low-dielectric (apolar) segments of the protein; green and yellow represent polar and apolar regions of the annular lipids around the protein. Numbers represent thicknesses and radii of different dielectric segments that undergo conformational changes. The red and blue lines depict putative equipotential surfaces around the protein and membrane under positive voltage in the outer (periplasmic) compartment relative to the cytoplasm. The shape of equipotential surfaces reflects the redistribution of electric field around the closed and open pore.

We hypothesize that MscL’s dipole arises from uneven charge distribution between the cytoplasmic and periplasmic domains, but the motion of these charges in the course of gating transitions (closed ↔ expanded ↔ open) is also accompanied by an electric field re-distribution due to protein flattening, pore expansion, hydration, and opening. Figure 4a visualizes the groups of charged residues and the distribution of electrostatic potential for *E*. *coli* MscL models in the closed and open states. The charged groups were taken in their default protonation states at neutral pH. The computation was done with the Particle Mesh Ewald (PME) algorithm in a vacuum, i.e., it reflects only the protein contribution to the electrostatics, unmitigated by the medium. The colour density indicates that the periplasmic side harbours the loops with the highest density of net negative charge, whereas the cytoplasmic side is richer in positive charges. These groups of charges clustered on opposite sides of the membrane form an effective dipole. We have to note, however, that all the smaller partial charges (e.g. hydroxyls), as well as the dipole moments of the transmembrane helices, also bring certain distributed contribution to the calculated net dipole moment. Approximating multiple partial charges and the resulting electrostatic field inside the protein with a single linear dipole is meant only to highlight the field density centres on the longitudinal cross-section of the system. This is a qualitative representation of the effective changes in charge and field distributions. The transition from the closed to the open state flattens the barrel essentially bringing the charges closer, but at the same time it brings the boundaries of the applied potential over the protein barrel closer together, thus increasing the intensity of the local transmembrane field. In addition, pore opening changes the distribution of electric field along the conductive pathway. The experiment (Fig. 1) instructs us that negative pipette potential (on the periplasmic side) makes opening of WT MscL somewhat less favourable. This seems counterintuitive because this voltage configuration should favour a flatter, i.e. open, conformation. However, changes in electric field distribution near the reshaping protein may reverse the effect. Indeed, if we consider the probable positions of equipotential surfaces around the closed and (flatter) open conformations, it may become possible that with opening the domains that used to extend beyond the charged regions now pack deeper into the membrane, as the structural models of channel opening suggest^18,19^, and the charged clusters constituting MscL’s dipole become exposed to a larger fraction of the external electric field that drops across the membrane segment harbouring the channel. In contrast, the taller and more ‘protein-shielded’ closed conformation (Fig. 4b, left) becomes more favourable at negative pipette potentials. We have constructed a cylindrical system consisting of the channel, lipid, and water parts, parametrized with “ballpark” geometric, dielectric and conductive properties approximating MscL. The cylinders represent the closed, expanded, and open states of WT and V23T MscL embedded into the lipid bilayer (the parameters and the details of calculations are provided in the Supplement.). The dimensions of the barrels, annular lipid ring, and electrostatic properties of the channel were based on the homology models of WT and V23T MscL equilibrated in the all-atom lipid bilayer and were rounded to the nearest 5 Å where possible. Some of the parameters such as diameters of the channels barrel in the closed and the open states, and septum thickness in the intermediate state of V23T had stronger effect on the estimated electrostatic energies and were ‘scanned’ with a 1 Å steps to reproduce the experimental data, while still conforming to the experimentally measured lateral expansion area and open pore conductance. Our order-of-magnitude calculations (Table 1) show that the observed energetic effects of the dipole component can be reasonably estimated even in simplified cylindrical representation of the channel (Fig. 4b).

**Table 1:** The dielectric (capacitive) and dipole contributions diminishing the energy of tension-driven closed-to-open transitions in WT and V23T MscL.

Conformation → Subsystem ↓		WT				V23T						
		Closed	Open	Open-Closed	Experiment	Closed	Expanded	Open	Expanded-Closed	Open-Closed	Effective Conducting-Closed	Experiment
	Pore radius, nm	**1**	**1**.**45**	**0**.**45**		**1**.**15**	**1**.**25**	**1**.**45**	**0**.**1**	**0**.**3**	**0**.**2**	
Capacitive energy, kT	Annular lipid ring	−0.15	−0.22	−0.07		−0.16	−0.21	−0.22	−0.05	−0.07	−0.06	
	Protein barrel	−0.30	−0.71	−0.40		−0.35	−0.67	−0.77	−0.32	−0.43	−0.37	
	Ion-conducting pore	−0.05	−0.91	−0.86		−0.08	−4.67	−0.91	−4.60	−0.84	−2.72	
	Total capacitive	−0.50	−1.84	−1.34	**−1**.**35**	−0.58	−5.55	−1.91	−4.97	−1.33	−3.15	**−3**.**11**
Dipole energy, kT		−29.33	−31.31	−1.98	**−1**.**93**	−29.10	−27.99	−31.31	1.11	−2.21	−0.55	**−0**.**53**
Total electrostatic energy, kT		−29.83	−33.15	−3.32	**−3**.**28**	−29.68	−33.54	−33.22	−3.86	−3.54	−3.70	**−3**.**64**

The contributions were calculated for the hyperpolarizing +100 mV pipette voltage and 300 K.

In contrast to the dipole effect, the dielectric effect tends to maximize the exposure of high-dielectric components to electric field as it happens when part of a protein in the pore is displaced and substituted by water. For a cylindrical capacitor of radius *r* and thickness *h* filled with a medium of dielectric permeability $\varepsilon$, the dielectric energy *E*_*c*_ can be expressed as a function of applied voltage:

5 $${E}_{c}=\frac{\varepsilon {\varepsilon }_{0}\pi {r}^{2}}{2h}{V}^{2}.$$

The total dielectric contribution to energy for a membrane-embedded MscL can be presented as three additive parts: *E*_*b*_ related to flattening of the protein barrel, *E*_*a*_ caused by distortion of annular lipids, and *E*_*i*_ representing dielectric polarization of water that displaces the protein and fills the open pore interior.

The reason for the capacitive (dielectric) energy change for WT MscL is predominantly explained by the replacement of the low-dielectric pore-occluding part of the protein in the closed conformation with a volume of water in the open pore polarized by the part of the external voltage that drops across the transmembrane conductive pathway. Table 1 presents the key geometric parameters of the transition for WT and V23T MscL, dielectric properties of system components, and calculated energies associated with the transitions.

### The tests for the local field effects introduced by membrane asymmetry

**Figure 5:**
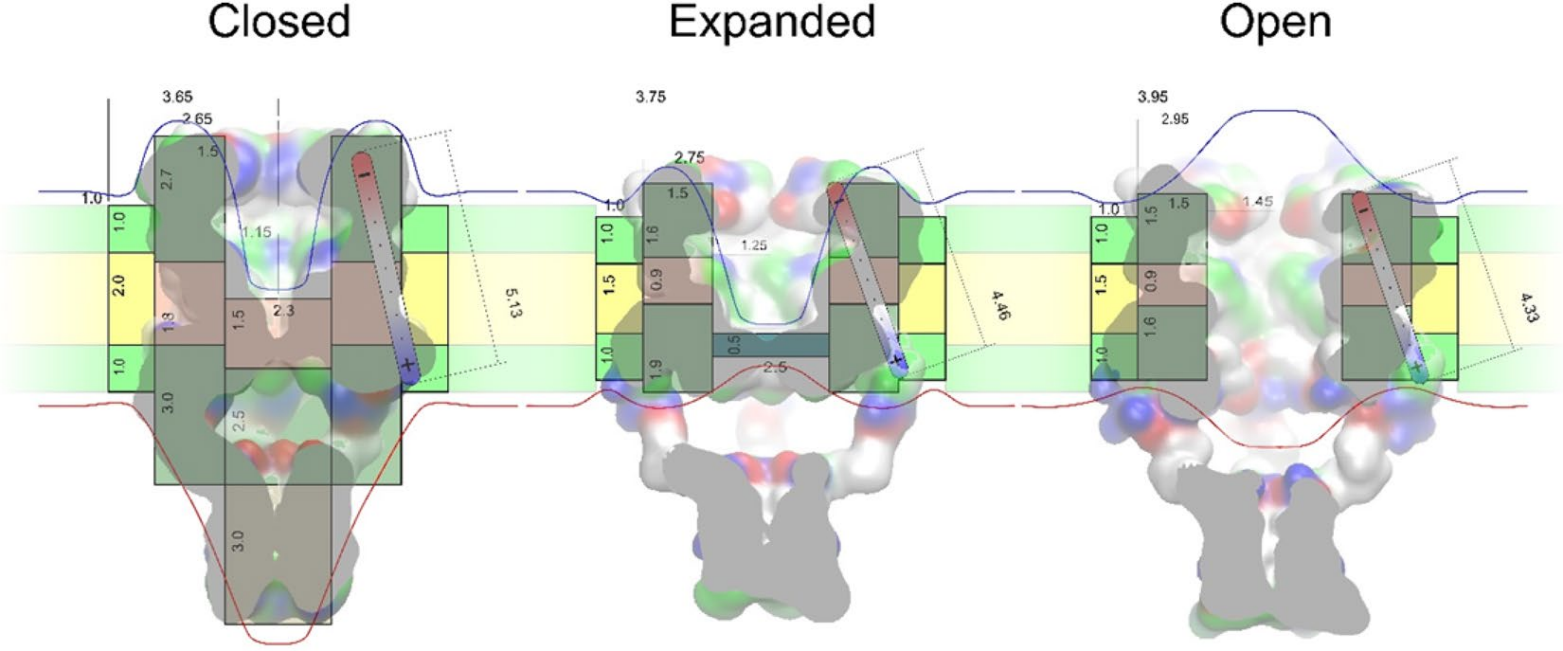
Estimations of V23T MscL’s changes in dipole and capacitive energies as a result of transitions to the expanded (subconductive) and open states. Simplified cylindrical representations of three main conformations of V23T MscL (closed, expanded, and open) overlaid on atomistic models of respective states presented as surfaces. Grey regions represent high-dielectric (polar) and pink represent low-dielectric (apolar) segments of the protein; cyan domain in the expanded (subconductive) state shows a polar (likely hydrated) occlusion of the pore; green and yellow represent polar and apolar regions of the annular lipids around the protein. Numbers represent approximate thicknesses and diameters of different dielectric segments that undergo conformational changes in nm. The red and blue lines depict equipotential surfaces around the protein and membrane, in this particular case blue line designates positive voltage in the outer (periplasmic) compartment.

The above results show that the energy of the MscL gating transition is affected by an externally applied electric field (Fig. 5). For V23T MscL the effect is especially strong because the geometric shape of this pre-hydrated channel in expanded/subconductive states, which is highly populated in V23T, permits ion penetration into the vestibule which concentrates the electric field on a relatively thin septum (Figs. 4 and 5). The septum is likely to be hydrated and it may take a minimal conformational transition that would generate a leakage, i.e., a subconductive state. The system subjected to voltage will likely favour subconductive states over the fully open state because in the latter case high pore conductance will ‘shunt’ the voltage across that narrow region thus reducing the local electric field. The voltage-dependence of V23T MscL is therefore likely imparted by a highly localized electric field. To test this notion and to answer the question of whether the channel feels not just the potential difference between the bulk solutions on the two sides of the membrane, but the superposition of both the external field and the intrinsic lipid-generated field, we studied voltage-dependent gating of V23T MscL in asymmetric DIBs. Such interfacial bilayers formed from lipid leaflets made of ester (DPhPC) and ether (DOPhPC) lipids bear a constant electrostatic bias created by the difference of interfacial dipole potentials of the two monolayers.

**Figure 6:**
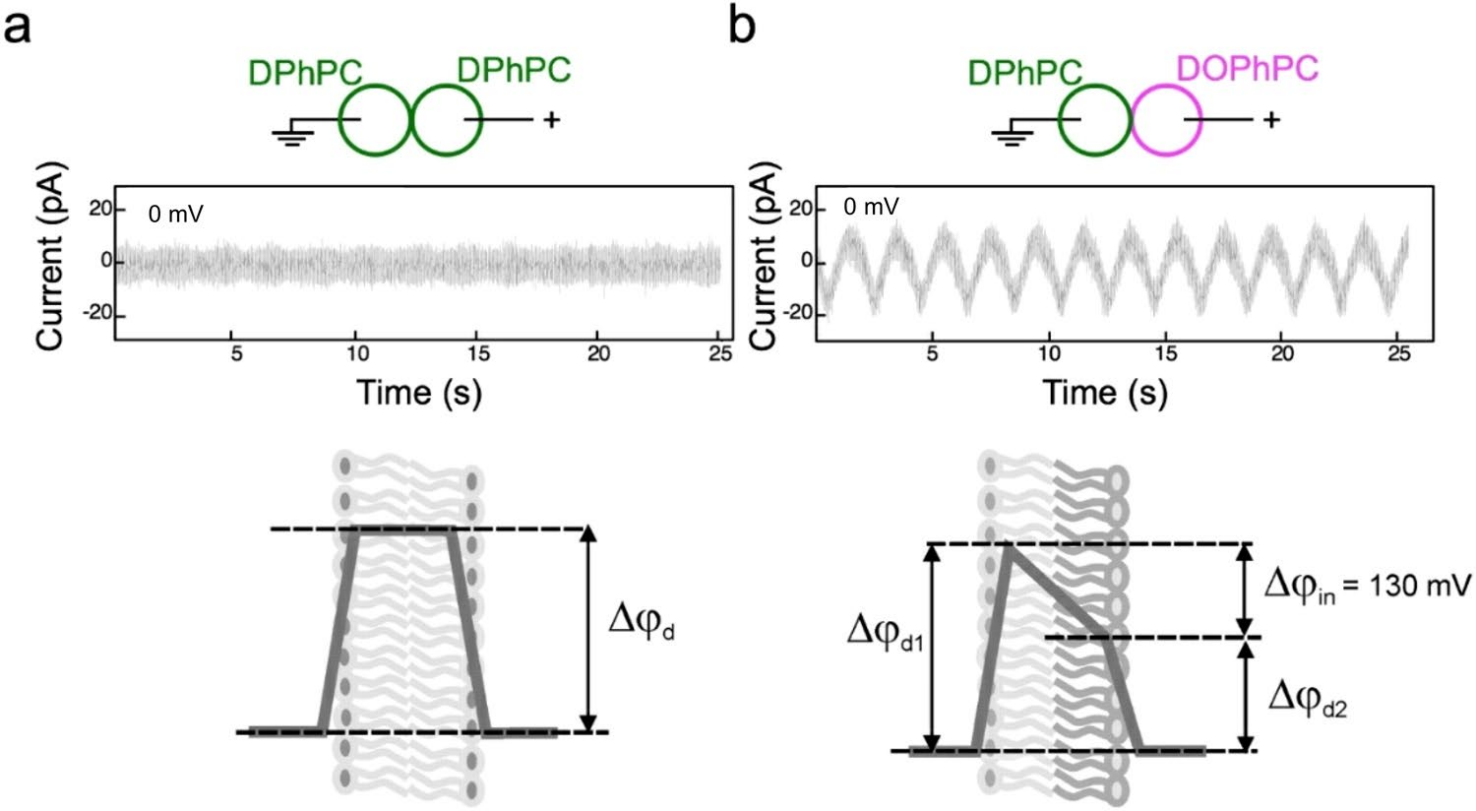
Displacement currents recorded on symmetric DPhPC/DPhPC and asymmetric DPhPC/DOPhPC DIBs in response to periodic mechanical oscillations. The external voltage was set to zero in both experiments, and the recordings were done in the absence of reconstituted channels.

Figure 6 illustrates the electrostatic effect of the permanent intrinsic electrical bias inside DIB revealed by mechanical oscillations periodically changing the area of the interface bilayer. Both traces in Fig. 6a,b are recorded under short-circuit conditions. When external voltage is 0, the displacement current predicted by the capacitive current formula ${I}_{c}={\rm{\Delta }}{{\rm{\varphi }}}_{{\rm{in}}}\times dC/dt$ is also zero in the case of a symmetric bilayer. When the bilayer is asymmetric and holds a permanent intrinsic bias, the current is detected. As was shown previously^14^, introduction of an external bias of ~130 mV compensates the intrinsic field in the asymmetric bilayer made of DPhPC and DOPhPC and therefore zeros the capacitive current.

**Figure 7:**
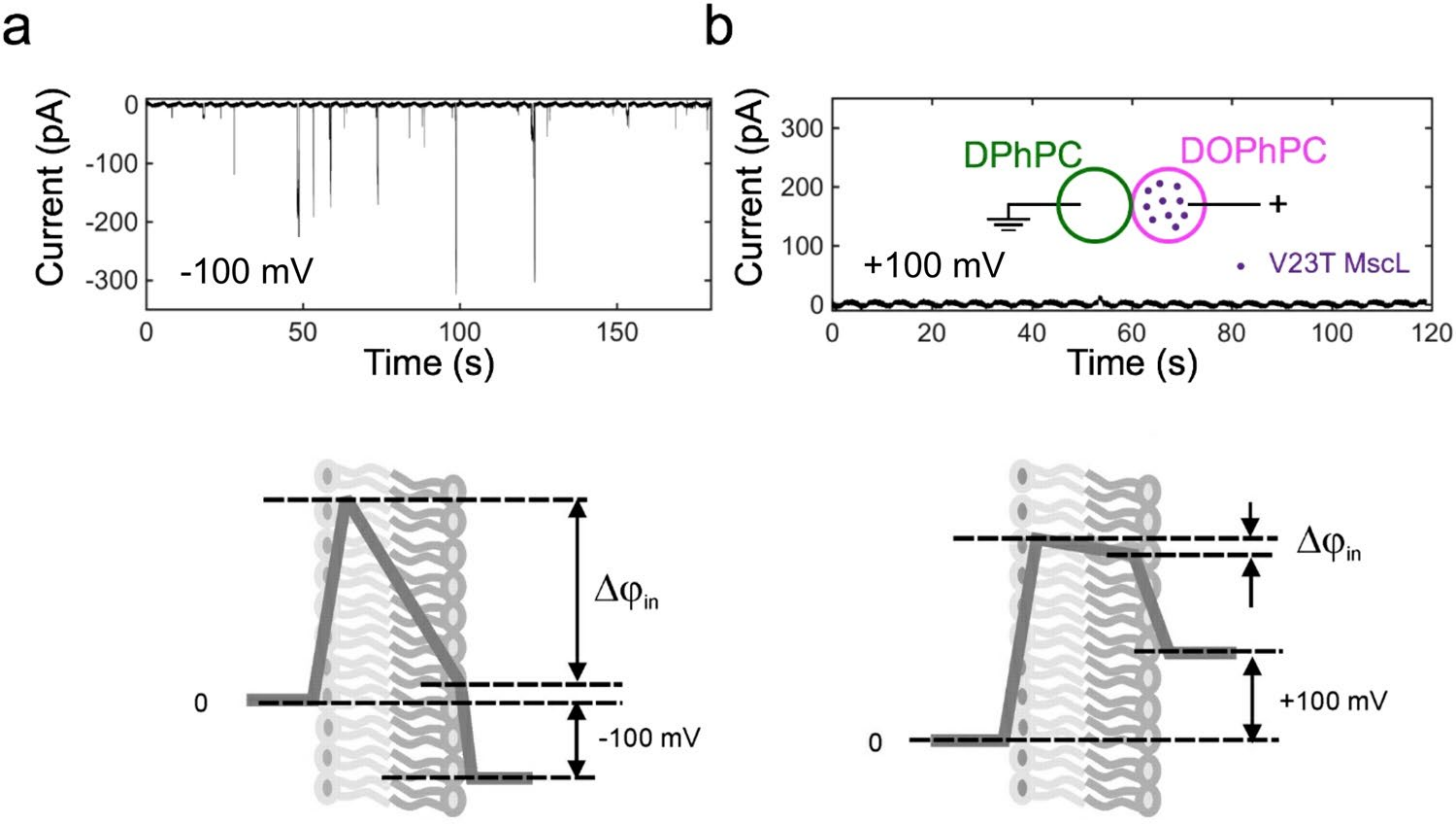
Activities of V23T MscL reconstituted into an asymmetric DIB folded from DPhPC (left) and DOPhPC (right). Cartoons below depict electric field distribution across electrically neutral asymmetric membranes composed of two leaflets characterized by different surface dipole potentials. External voltage of −100 mV amplifies the internal potential Δφ_in_, promoting massive gating of V23T MscL. Positive +100 mV potential negates the internal potential drop and V23T MscL remains silent.

Figure 7 shows traces recorded in an asymmetric DPhPC/DOPhPC bilayer with directionally reconstituted V23T MscL. The channel is highly active at −100 mV and completely silent at +100 mV. This is a stark inversion of voltage dependence observed in symmetric DIBs (Fig. 3). The explanation comes from the consideration of electric potential profiles across asymmetric membranes given at the bottom of Fig. 7. The intrinsic bias of approximately 130 mV, which exists at zero applied voltage (Fig. 6b), is differentially influenced by the external voltage. The intramembrane voltage drop is amplified by negative (−100 mV) potential applied to the cis compartment and the channel gates with higher probability to higher conductive levels. The application of a positive 100 mV largely negates the already existing −130mV down to a −30 mV (the sum of both the externally applied voltage and the intrinsic bias), producing a value proven insufficient for the activation of V23T MscL in DIBs^15^. Therefore, the open probability of V23T MscL under given tension is augmented by the local potential dropping across the central part of the membrane between the boundary layers.

## Discussion

MscL’s transmembrane domain is composed of five pairs of interlocked TM1-TM2 helices (Fig. 4a). When subjected to membrane tension, the barrel expands in an iris-like manner through tilting of helical pairs^18–20^. The expansion of the barrel of WT MscL proceeds through a series of non-conductive conformations reaching the transition barrier positioned at about 70% on the in-plane expansion coordinate (ΔA) between the closed and fully open states^5^. The position and height of the rate-limiting barrier are apparently defined by the properties of the hydrophobic constriction lined by the L19 and V23 sidechains contributed from all five subunits. In the closed state, the constriction appears completely dehydrated (vapour-locked) and therefore leak-proof^4^. Increased tension produces more expanded states in which hydration of the hydrophobic constriction eventually becomes favourable^21^, thus creating a ‘tipping point’ on the energy profile leading to the open state^4^. The total transition energy includes terms related to the changes in protein-protein contacts, solvation, stretching of the periplasmic loops^22^, as well as transversal compression and bending of the annular lipids around the protein since the tilting of helices is accompanied by a substantial flattening of the barrel^23,24^.

Solvation of the initially dehydrated hydrophobic pore stands out as a decisive factor since hydrophilic substitutions in that region dramatically decrease both the barrier and the energy difference between the closed and open states^3,4,25,26^. While the cooperativity of WT MscL gating is defined apparently by a single hydration event followed by essentially complete opening, the pre-hydrated pore of the V23T mutant allows for partial openings manifested as multiple subconductive states. The hydrophilic pore in the mutant is also pre-expanded in the resting state, and for this reason the apparent total lateral expansion is smaller than in the WT (18 nm^2^ versus 20 nm^2^). This initial volume of water stored deep in the pore may serve as the nucleation site for stronger hydration favoured by the applied transmembrane voltage. Indeed, water as a component with the highest dielectric constant in our system can be pulled into the region of strong electric field^27^ located in the pore constriction near the middle of the membrane. Importantly, the voltage threshold at which the electrically-induced pores start accumulating in the lipid bilayer is in the range of 150–200 mV^28^, and the size of these pores is estimated to be comparable to MscL diameter (~ 3–5 nm)^29–33^. It appears that the effect of water treeing in cable insulators has precisely the same nature^34^.

**Figure 8:**
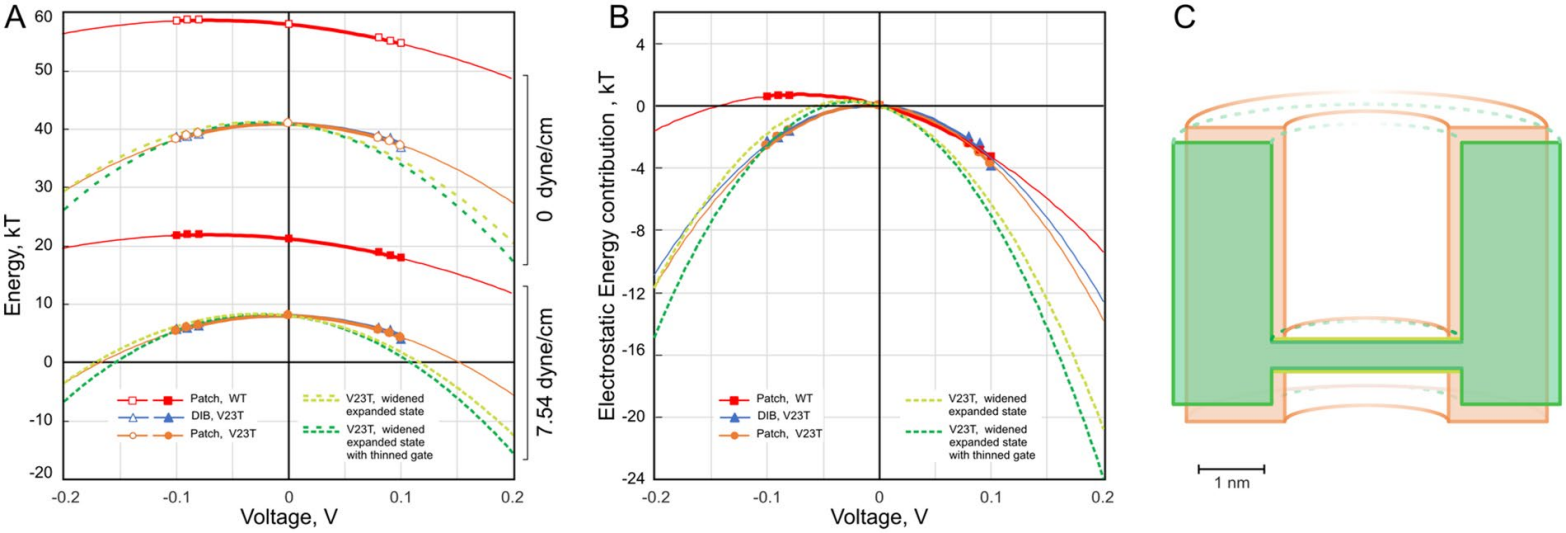
The summary of voltage effects on the transition energy of WT (red), V23T (orange, blue) and a hypothetical mutant with a slightly wider barrel in the expanded state (light green) and also thinned gate (dark green) extrapolated to ±200 mV (pipette). The positive voltage in this plot corresponds to hyperpolarization. (**A**) Energies for the opening transition presented in the kT scale for WT and V23T at 0 tension (upper curves) and for the tension of 7.54 mN/m (lower curves). (**B**) The comparison of slopes of electrostatic contributions when the origins of all curves are rescaled to zero. (**C**) The cartoon illustrating the effective geometry of voltage-sensitive V23T MscL in the expanded subconductive state (orange) and the shape of a hypothetical re-engineered channel characterized by an open-like pore width and slightly thinned hydrophilic septum separating the compartments (green).

The summary of gating energetics as a function of voltage for WT and V23T MscL is shown in Fig. 8A at two tensions. Since the voltage range accessible for patch-clamp or DIBs experiments is about ±120 mV, to illustrate expected energies in a fully energized state of the membrane^35,36^ we extrapolated curves to ±200 mV. The top curve on panel a reflects the total opening energy (*E*_*o*_) for the WT, which peaks at 58 kT at zero tension and zero voltage and asymmetrically bends down at ±200 mV. The result shows that WT is only slightly sensitive to voltage and will never open by physiological membrane potential at zero tension. To illustrate the scale of tension effect on the gating energy, we chose γ = 7.54 mN/m, corresponding to the peak tension in DIBs experiments (Fig. 2c,d). We should note that this tension is considerably below the WT activation threshold, however it does already shift the curve down to a new position with the energy maximum at 22 kT. This energy decrease (~36 kT) is due to the γΔA term, which reflects the WT MscL cooperative transition from the closed to the fully open state associated with the ΔA of 20 nm^2^. The electric contribution, responsible for bending the curve down at both ends, remains the same and extrapolations predict that at 7.54 mN/m membrane hyperpolarization to 200 mV would bring *E*_*o*_ down to ~12 kT. This energy sets the open probability to ~10^−5^ and thus may produce occasional openings.

For V23T MscL, the zero-tension *E*_*o*_ has its maximum at 41 kT^3^, and the dielectric contribution produces a symmetrical parabolic energy dependence, which bends the curve down more substantially, reaching 29 kT at the ends. At γ = 7.54 mN/m, the entire curve slides down by γΔA = 33 kT, approximating the energy change due to opening to either a subconductive or fully open state with equal probability^3^. At this tension, the energy curve has its maximum at 8 kT, and the parabolas extrapolated to +200 mV predict that the transition energy may drop down to −5 kT, driving the channel conductive most of the time (99%) which, even at low-conductive substates, will become dangerous for the cell energetics. The zoomed-up view of electric field contributions for all energy curves with origins re-scaled to zero is presented on Fig. 8B. These effects that are negligible at low voltages typically used in electrophysiological recording (20–30 mV), become more pronounced at potentials typical for a fully energized state of the bacterial membrane (150–200 mV)^35^.

It is very feasible that the amino acid sequence of MscL can be further modified to design a channel that is even more sensitive to voltage than the V23T mutant. The green curves on Fig. 8 represent a hypothetical situation assuming that we can re-engineer the channel such that the barrel in the expanded state becomes 16% wider than the one in V23T (i.e. reaches the width similar to that of the fully open state, while the gate still remains impermeable to ions). This increases the volume of polarized water in the pore and further reduces the transition energy at +200 mV, which now becomes negative. This will drive spontaneous expansion and opening events even at no tension. Thinning of the partially permeable septum separating the upper and lower compartments by only 1 Å (20%) will further decrease the energy providing additional stabilization of the subconductive state.

The data presented in Fig. 7 show that the dielectric energy of water stored in the hydrated pore of V23T MscL is determined by the superposition of the external field and the drops of dipole potential across the interfacial layers. This provides a useful tool for the introduction of a constant field that may bias positions and sensitivities of membrane-embedded voltage sensors. In our case, this sensor is the water residing in the pore.

The data presented here depicts a consistent mechanistic picture that WT MscL gates between the open and closed states separated by an unusually large energy gap, ensuring that this osmotic safety valve stays shut to near lytic tension for the bacterial inner membrane. The overcoming of the large energy gap seems to involve a pseudo-phase transition at the nanoscale as a large pore is wetted. The effect of the large (physiological) bacterial membrane voltage appears to be minimized by two factors: (1) the ‘dry’ pore which essentially excludes water from penetrating and changing of dielectric properties inside the membrane, and (2) likely compensation of intrinsic protein electrostatics by the surrounding components.

Our first quantitative dataset shows that under normal voltages across the energized cytoplasmic membrane WT MscL resists spurious openings, and thus, is capable of preserving the barrier function of the inner membrane and bacterial energetics. The valine-to-threonine substitution in the constriction facilitates an opening transition and makes the channel significantly more prone to occupy subconductive states. This occurs at lower tensions when voltage approaches levels of a normally energized membrane. This substitution changes the character of voltage dependence, which is consistent with the primary role of water filling the pore and acting as a high-dielectric medium swelling the pore. This substitution makes the channel slightly toxic (as it dissipates transmembrane gradients) and for this reason polar substitutions for valine in this position are never found in any of the MscL orthologs. Threonine attracts water into the desolvated pore constriction and thus compromises the ability of the channel to hold high membrane potentials. However, the physical principle of MscL voltage modulation by a polar solvent can potentially be used for the engineering of molecular devices enabling us to adjust the tension sensitivity of sensors by voltage. Another notable aspect of this work is that here we provide the very first description of a surface dipole effect on a reconstituted channel imparted by the asymmetric lipid environment. This bears significance for both biology and bioinspired engineering because this result emphasizes the roles of interfacial dipole layers in setting the local electric field for the embedded molecule.

## Methods

The patch-clamp experiments were performed as described previously^3,4,6^. The giant *E*. *coli* spheroplasts were generated from MJF465 *E*. *coli* cells^7^ expressing either WT of V23T MscL. Patches were formed in symmetric 200 mM KCl, 90 mM MgCl_2_ and 10 mM CaCl_2_ in 10 mM Hepes-KOH buffer (10 mM, pH7.4). All recordings were done in excised inside-out patches under linear pressure ramps delivered from a modified HSPC-1 pressure clamp apparatus (ALA) using Axopatch 200B amplifier and PClamp 10 software.

The droplet interface bilayer (DIBs) recordings under periodic mechanical stimulation were performed exactly as in Ref.^14,15^. The aqueous droplets consist of a liposome suspension (2 mg/ml of either DPhPC or DOPhPC, Avanti Lipids), 500 mM of potassium chloride (KCl, Sigma Aldrich), and 10 mM of 3-(N-morpholino)propanesulfonic acid (MOPS, Sigma Aldrich) in deionized water (>18.2 MΩ.cm), pH 7. Hexadecane (99%, Sigma) is used as the oil phase. The hydrogel phase, used in the glass capillaries, consist of 40% (w/v) PEG-DMA contains 0.5% (w/v) Irgacure 2959, and is mixed with a 500 mM KCl and 10 mM MOPS, pH 7 electrolyte solution. The liposome and hydrogel solutions are prepared and stored as described elsewhere^37^. V23T MscL mutants, first generated and characterized by Anishkin *et al*.^3^, were isolated, reconstituted, and stored as described elsewhere^15,37^.

The DIB test setup is built and used as described by Najem *et al*.^15,37^. In the case of asymmetric studies, the droplet connected to the headstage contained DOPhPC, while the one connected to the ground contained DPhPC; salt concentration in both droplets were the same (500 mM KCl). We used Axopatch 200B and Digidata 1440 A (Molecular devices) in voltage-clamp mode for our electrical recording as described elsewhere^15,37^. The patch-clamp experiments were conducted as described elsewhere^3,6,18^.

The open probability of WT and V23T MscL in the course of the ramp stimulation in patch clamp was calculated as the fractional population current relative to the current at saturating pressure. In the case of DIBs experiments while we often deal with incomplete openings of single V23T channels to subconductive states, we still used the same approach of the fractional integral current to be compatible with patch-clamp estimates. For DIBs, the fractional population current was estimated as product of the average fraction of the time the channels were observed fully or partially open and the average relative single-channel conductance (as a fraction of a fully open single-channel current). It should be noted, that while the published experimentally estimated expansion area of V23T MscL from the closed to the fully open state (18 nm^2^) is only slightly smaller than that of WT MscL (20 nm^2^), the abundance of sub-conductive states with smaller expansion areas and lower energy costs^3^ creates a spread of the conductive events towards the lower tension values. This creates shallower integral activation curve and an appearance of a smaller expansion area, resembling the effect of heterogeneity in the channel population^6^.

## Electronic supplementary material

Supplementary Information

## Data Availability

All data generated or analyzed during this study are included in the published article. Original data files are available upon request as needed.
