# Supplementary material for: The voltage-dependence of MscL has dipolar and dielectric contributions and is governed by local intramembrane electric field
Source: Sci Rep. 2018 Sep 11;8:13607. doi: 10.1038/s41598-018-31945-x (PMC6133944; doi:10.1038/s41598-018-31945-x)
Supplement: Supplementary file 1 — Supplementary Information [file 41598_2018_31945_MOESM1_ESM.pdf]

The voltage-dependence of MscL has dipolar and dielectric contributions and is governed by local intramembrane electric field

Joseph S. Najem[1,2], Ian Rowe[3], Andriy Anishkin[3], Donald J. Leo[4], and Sergei Sukharev[3,*]

[1]Joint Institute for Biological Sciences, Oak Ridge National Laboratory, Oak Ridge, Tennessee 37830, United States; [2]Department of Mechanical, Aerospace, and Biomedical Engineering, University of Tennessee, Knoxville, Tennessee 37916, United States; [3]Department of Biology, University of Maryland, College Park, MD 20817, United States; [4]College of Engineering, University of Georgia, Athens, Georgia 30605, United States.

[*]Corresponding Author:

Sergei Sukharev, Department of Biology, University of Maryland, 3216 Biology-Psychology Bldg., College Park, Maryland 20742, United States, email: sukharev@umd.edu, phone: 301-405-6923

Contributions of the dipole and capacitive components to the energy of MscL channel in the electric field at the positive potential

The purpose of these calculations is a qualitative estimate for the voltage-dependent energy contributions of different parts of the MscL-lipid system to the total energy cost of channel gating. While there are numerous simplifications to make the calculations straightforward, we believe the results grasp the essential trends in the electrostatic energy contributions during the gating transitions of MscL.

All the estimations were made in a continuum-dielectric simplification, with MscL channel and annular lipids represented as a set of concentric cylinders of a certain dielectric permeability (based on the typical values for polar and non-polar regions of proteins and lipid bilayer, see "Constants and material properties" section of this supplement), which streamlines the calculations of the capacitive energies.

The cylindrical geometry of the channel in the closed, expanded, and open states was roughly approximated from our published homology models for WT MscL and the models for V23T MscL developed using them as a template. For each model, the channel is represented as two concentric cylinders:
1) the inner cylinder represents the pore part.
For the closed state, it consists of the hydrophobic gate on the periplasmic side, hydrophilic region at the cytoplasmic side of the gate, and relatively hydrophobic cytoplasmic bundle formed by C-terminal residues.
For the expanded state (prominent only in V23T MscL), the pore cylinder is only a slim hydrophilic constriction formed by a hydrated polar residues. It prevents the passage of ions, but has high dielectric permeability.
For the fully open state, the inner cylinder is ion-conductive and completely filled by water.
2) the outside protein cylinder represents the transmembrane protein barrel.
It consists of three layers - polar periplasmic and cytoplasmic parts, with non-polar layer in between. This architecture is common for all the conformational states, however the cylinder decreases in height and increases in width as channel opens.
Lipid bilayer is modeled as two domains:
1) a cylinder of annular lipids surrounding the channel barrel. It has three layers - polar headgroup layers at the periplasmic and cytoplasmic sides and non-polar core of the lipid tails. The cylinders gets thinner on channels expansion and flattening.
2) a stable bulk membrane. It consists of the three layers as well (polar headgroups an non-polar core), however it remains stable through the whole conformational cycle, and therefore it was not included in calculations of the electrostatic energy changes.

The specific spatial dimensions for each model are specified in "Geometry of the annular lipids ring", "Geometry of the protein barrel", and "Geometry of the ion- conducting pore" subsections of the supplement, and visualized on the embedded images (all the distances are in nm).

The dipole moment of the transmembrane part of the channel was approximated as two charges located at the maximum and minimum of the electrostatic field calculated separately for each model in all-atom representation using PME (Particle Mesh Ewald) Electrostatics plugin of VMD, and overlaid on the cylindrical models. The values specific to each model can be found in "Geometry of the dipole in the external electric field" sections.

The applied electric field was assumed to have 90% drop of the external bulk values between the cytoplasmic and periplasmic surfaces of the protein or lipid (reflected in parameter "U" in the "Experimental Conditions" section. For the cylindrical representation, the equipotential levels at these surfaces were approximated by cubic splines smoothly connecting the points in the bulk at ~1 Angstrom distance from the centers of the protein or the lipid surfaces (see the images for visual representations). The intermediate potential levels were derived through linear interpolation between the potential boundaries and color -coded on the images from positive (blue) to negative (red).

For every model, the capacitive contribution to the electrostatic energy is detailed in it's "Capacitors' energy" section. The energy of each cylindrical part was estimate as an energy of a consecutive set of three cylindrical capacitors with the same area but different thickness and dielectric permeability (e.g. layers of polar headgroups and the non-polar core of the annular lipids cylinder). The contributions of all the concentric cylindrical parts (pore, barrel, and annular lipids) were added together for the net capacitive energy of a particular conformation.

The electrostatic contribution of the dipole of the protein barrel was then summed up with the capacitive contribution, taking into account the directionality of the field (i.e. the dipole part was favorable at the voltages with the positive potential at the periplasmic side, and unfavorable with the negative).

For all the models, the capacitive and dielectric components are integrated and compared to experimental data in the "Summary for all the models" section at the end of the supplement.

Constants and material properties

$\varepsilon_0 := 8.85 \cdot 10^{-12} \frac{F}{m}$	Dielectric permeability of vacuum
$\varepsilon_w := 80.1$	Relative permittivity of water
$\varepsilon_{mp} := 20$	Relative permittivity of the polar part of the membrane
$\varepsilon_{mn} := 2$	Relative permittivity of the nonpolar part of the membrane
$\varepsilon_{pp} := 30$	Relative permittivity of the polar part of the protein
$\varepsilon_{pn} := 7$	Relative permittivity of the nonpolar part of the protein
$\varepsilon_{pc} := 18$	Relative permittivity of the C-terminal bundle of the protein
$\varepsilon_{pg} := 55$	Relative permittivity of the expanded gate in V23T
$f_m := 0.9$	Fraction of the bulk membrane potential that drops "surface-to-surface" on protein and lipid.

Experimental conditions

$T := 300\ K$	Temperature
$\gamma := 10 \frac{dyne}{cm}$	Membrane tension
$U_e := 100\ mV$	Applied external transmembrane potential (pipette/periplasmic site)
$U := f_m \cdot U_e = 90\ mV$	Drop of the external transmembrane potential at the protein and lipid boundaries

Model parameters (WT MscL)

$Eo := 58\,k \cdot T = 2.4023 \cdot 10^{-19}\,J$ Open-closed energy difference

$\Delta A := 20\,nm^2$ Open-closed expansion area

Electrostatic contribution for the Closed conformation

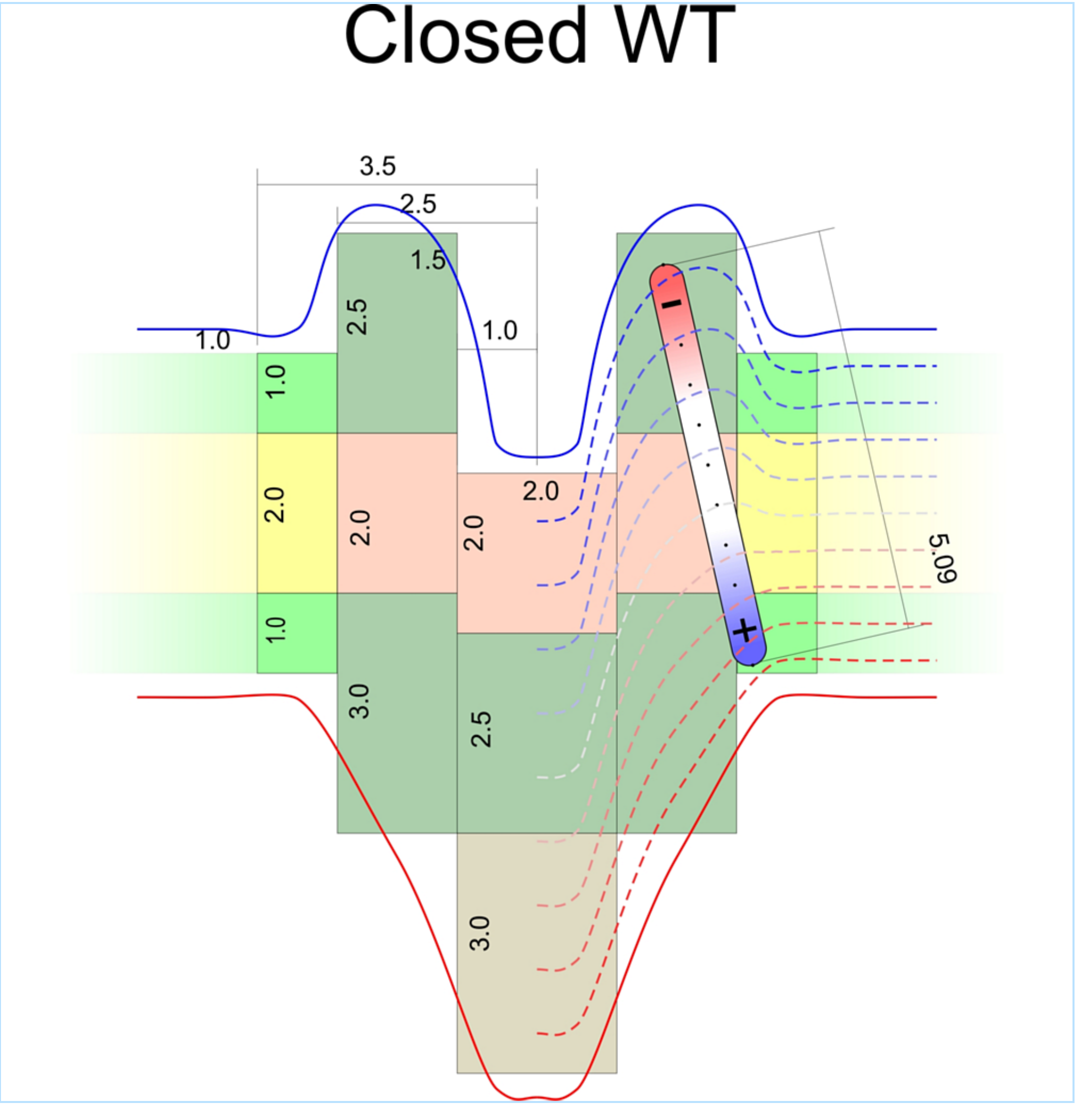

Geometry of the annular lipids ring

$r_a := 3.5\,nm$ External radius of the ring

$h_{ap} := 1.0\,nm$ Height of the polar region of the ring at the periplasmic side

$h_{an} := 2.0\,nm$ Height of the nonpolar region of the ring

$h_{ac} := 1.0\,nm$ Height of the polar region of the ring at the cytoplasmic side

Geometry of the protein barrel

$r_b := 2.5\,nm$ External radius of the protein barrel

$h_{bp} := 2.5\,nm$ Height of the polar region at the periplasmic side

$h_{bn} := 2.0\,nm$ Height of the nonpolar region of the barrel

$h_{bc} := 3.0\,nm$ Height of the polar region at the cytoplasmic side

Geometry of the ion-conucting pore

$r_i := 1.0\ \mathrm{nm}$ Pore radius

$h_{in} := 2.0\ \mathrm{nm}$ Height of the nonpolar gate region

$h_{ip} := 2.5\ \mathrm{nm}$ Height of the polar region under the gate

$h_{ic} := 3.0\ \mathrm{nm}$ Height of the C-terminal bundle

Geometry of the dipole in the external electric field

$p := 2675\ \mathrm{D}$ Dipole moment of the channel

$d := 5.09\ \mathrm{nm}$ Length of the channel dipole

$f_d := 1.0$ Fraction of the dipole moment in the external electric field

$f_{en} := 0.92$ Fraction of the electric field where the negative charge is

$f_{ep} := 0.15$ Fraction of the electric field where the positive charge is

Capacitors' energy

$$C_a := \frac{\varepsilon_0 \cdot \pi \cdot \left(r_a^{\,2} - r_b^{\,2}\right)}{\frac{h_{ap}}{\varepsilon_{mp}} + \frac{h_{an}}{\varepsilon_{mn}} + \frac{h_{ac}}{\varepsilon_{mp}}} = 1.5165 \cdot 10^{-19}\ \mathrm{F}$$

Capacitance of the annular lipid ring

$$Ec_a := -\frac{C_a \cdot U^2}{2} = -0.1483\ \mathrm{k} \cdot T$$

Capacitive energy of the annular lipid ring

$$C_b := \frac{\varepsilon_0 \cdot \pi \cdot \left(r_b^{\,2} - r_i^{\,2}\right)}{\frac{h_{bp}}{\varepsilon_{pp}} + \frac{h_{bn}}{\varepsilon_{pn}} + \frac{h_{bc}}{\varepsilon_{pp}}} = 3.112 \cdot 10^{-19}\ \mathrm{F}$$

Capacitance of the protein barrel

$$Ec_b := -\frac{C_b \cdot U^2}{2} = -0.3043\ \mathrm{k} \cdot T$$

Capacitive energy of the protein barrel

$$C_i := \frac{\varepsilon_0 \cdot \pi \cdot r_i^{\,2}}{\frac{h_{in}}{\varepsilon_{pn}} + \frac{h_{ip}}{\varepsilon_{pp}} + \frac{h_{ic}}{\varepsilon_{pc}}} = 5.1899 \cdot 10^{-20}\ \mathrm{F}$$

Capacitance of the ion-conducting pore

$$Ec_i := -\frac{C_i \cdot U^2}{2} = -0.0507\ \mathrm{k} \cdot T$$

Capacitive energy of the ion-conducting pore

$$Ec := Ec_a + Ec_b + Ec_i = -0.5033\ \mathrm{k} \cdot T$$

Capacitive energy of the whole system

$$Ec_{closed} := Ec = -2.0847 \cdot 10^{-21}\ \mathrm{J}$$

Same value assigned to the closed state

closed

Dipole's_energy

$q_d := \frac{p}{d} = 1.753 \cdot 10^{-18}$ C

$q_d = 10.9415$ e

Effective charge of the dipole

$Ed := f_d \cdot q_d \cdot U \cdot (f_{ep} - f_{en}) = -1.2148 \cdot 10^{-19}$ J

$Ed = -29.3301$ k·T

Energy of the dipole part exposed to the gradient of the potential

$Ed_{closed} := Ed = -1.2148 \cdot 10^{-19}$ J

Same value assigned to the closed state

Capacitive contribution for the Open conformation

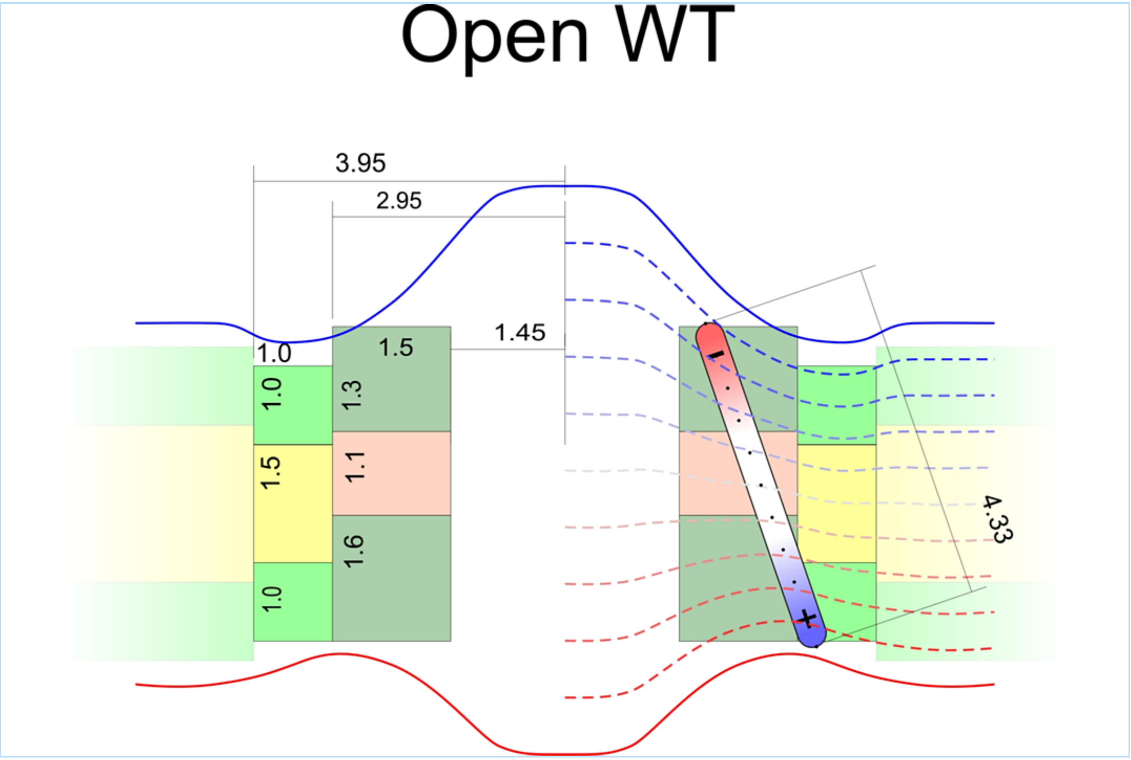

Geometry of the annular lipids ring

$r_a := 3.95$ nm External radius of the ring

$h_{ap} := 1.0$ nm Height of the polar region of the ring at the periplasmic side

$h_{an} := 1.5$ nm Height of the nonpolar region of the ring

$h_{ac} := 1.0$ nm Height of the polar region of the ring at the cytoplasmic side

Geometry of the protein barrel

$r_b := 2.95$ nm External radius of the protein barrel

$h_{bp} := 1.3$ nm Height of the polar region at the periplasmic side

$h_{bn} := 1.1$ nm Height of the nonpolar region of the barrel

$h_{bc} := 1.6$ nm Height of the polar region at the cytoplasmic side

Geometry of the ion-conucting pore

$r_i := 1.45$ nm Pore radius

Geometry of the dipole in the external electric field

$p := 2175$ D Dipole moment of the channel

$d := 4.33$ nm Length of the channel dipole

$f_d := 1.0$ Fraction of the dipole moment in the external electric field

$f_{en} := 0.89$ Fraction of the electric field where the negative charge is

$f_{ep} := 0.03$ Fraction of the electric field where the positive charge is

Capacitors' energy

$$C_a := \frac{\varepsilon_0 \cdot \pi \cdot \left(r_a^{\,2} - r_b^{\,2}\right)}{\frac{h_{ap}}{\varepsilon_{mp}} + \frac{h_{an}}{\varepsilon_{mn}} + \frac{h_{ac}}{\varepsilon_{mp}}} = 2.257 \cdot 10^{-19}\ \mathrm{F}$$

Capacitance of the annular lipid ring

$$Ec_a := -\frac{C_a \cdot U^2}{2} = -0.2207\ \mathrm{k} \cdot T$$

Capacitive energy of the annular lipid ring

$$C_b := \frac{\varepsilon_0 \cdot \pi \cdot \left(r_b^{\,2} - r_i^{\,2}\right)}{\frac{h_{bp}}{\varepsilon_{pp}} + \frac{h_{bn}}{\varepsilon_{pn}} + \frac{h_{bc}}{\varepsilon_{pp}}} = 7.2298 \cdot 10^{-19}\ \mathrm{F}$$

Capacitance of the protein barrel

$$Ec_b := -\frac{C_b \cdot U^2}{2} = -0.7069\ \mathrm{k} \cdot T$$

Capacitive energy of the protein barrel

$$\frac{U_{pore}}{U} = \frac{R_{pore}}{R_{pore} + R_{access}} \qquad \frac{R_{pore}}{R_{pore} + R_{access}} = \frac{\frac{d}{\rho \cdot \pi \cdot r^2}}{\frac{d}{\rho \cdot \pi \cdot r^2} + \frac{2}{4 \cdot \rho \cdot r}} \qquad \frac{U_{pore}}{U} = \frac{1}{1 + \frac{r}{2 \cdot d}}$$

$$U_{pore} := \frac{U}{1 + \frac{r_i}{2 \cdot \left(h_{bp} + h_{bn} + h_{bc}\right)}} = 76.1905\ \mathrm{mV}$$

Voltage dropping on the pore, based on Hall's equation

$$\frac{U_{pore}}{U} = 84.6561\ \%$$

Fraction of voltage dropping on the pore

$$U_{access} := \frac{U - U_{pore}}{2} = 6.9048\ \mathrm{mV}$$

Voltage dropping on the access region at one side of the pore

$$C_i := \frac{\varepsilon_w \cdot \varepsilon_0 \cdot \pi \cdot r_i^{\,2}}{h_{bp} + h_{bn} + h_{bc}} = 1.1706 \cdot 10^{-18}\ \mathrm{F}$$

Capacitance of the ion-conducting pore

$$Ec_i := -\frac{C_i \cdot U_{pore}^{\,2}}{2} = -0.8203\ \mathrm{k} \cdot T$$

Capacitive energy of the ion-conducting pore

$$C_{i\_access} := 8 \cdot \varepsilon_w \cdot \varepsilon_0 \cdot r_i = 8.2231 \cdot 10^{-18}\ \mathrm{F}$$

Capacitance of one access region of the ion-conducting pore

$$Ec_{i\_access} := -\frac{C_{i\_access} \cdot U_{access}^{\,2}}{2} = -0.0473\ \mathrm{k} \cdot T$$

Capacitive energy of one access region of the ion-conducting pore

$$Ec := Ec_a + Ec_b + Ec_i + 2 \cdot Ec_{i\_access} = -1.8426\ \mathrm{k} \cdot T$$

Capacitive energy of the whole system

$$Ec_{open} := Ec = -7.6318 \cdot 10^{-21}\ \mathrm{J}$$

Same value assigned to the open state

Dipole's energy

$q_d := \frac{p}{d} = 1.6755 \cdot 10^{-18}\ \mathrm{C}$ $q_d = 10.4578\ \mathrm{e}$ Effective charge of the dipole

$Ed := f_d \cdot q_d \cdot U \cdot (f_{ep} - f_{en}) = -1.2969 \cdot 10^{-19}\ \mathrm{J}$ $Ed = -31.3103\ \mathrm{k} \cdot T$ Energy of the dipole part exposed to the gradient of the potential

$Ed_{open} := Ed = -1.2969 \cdot 10^{-19}\ \mathrm{J}$ Same value assigned to the open state

---

Model parameters (V23T MscL)

$Eo := 41\,k \cdot T = 1.6982 \cdot 10^{-19}\ J$ Open-closed energy difference

$\Delta A := 18\ nm^2$ Open-closed expansion area

Capacitive contribution for the Closed conformation

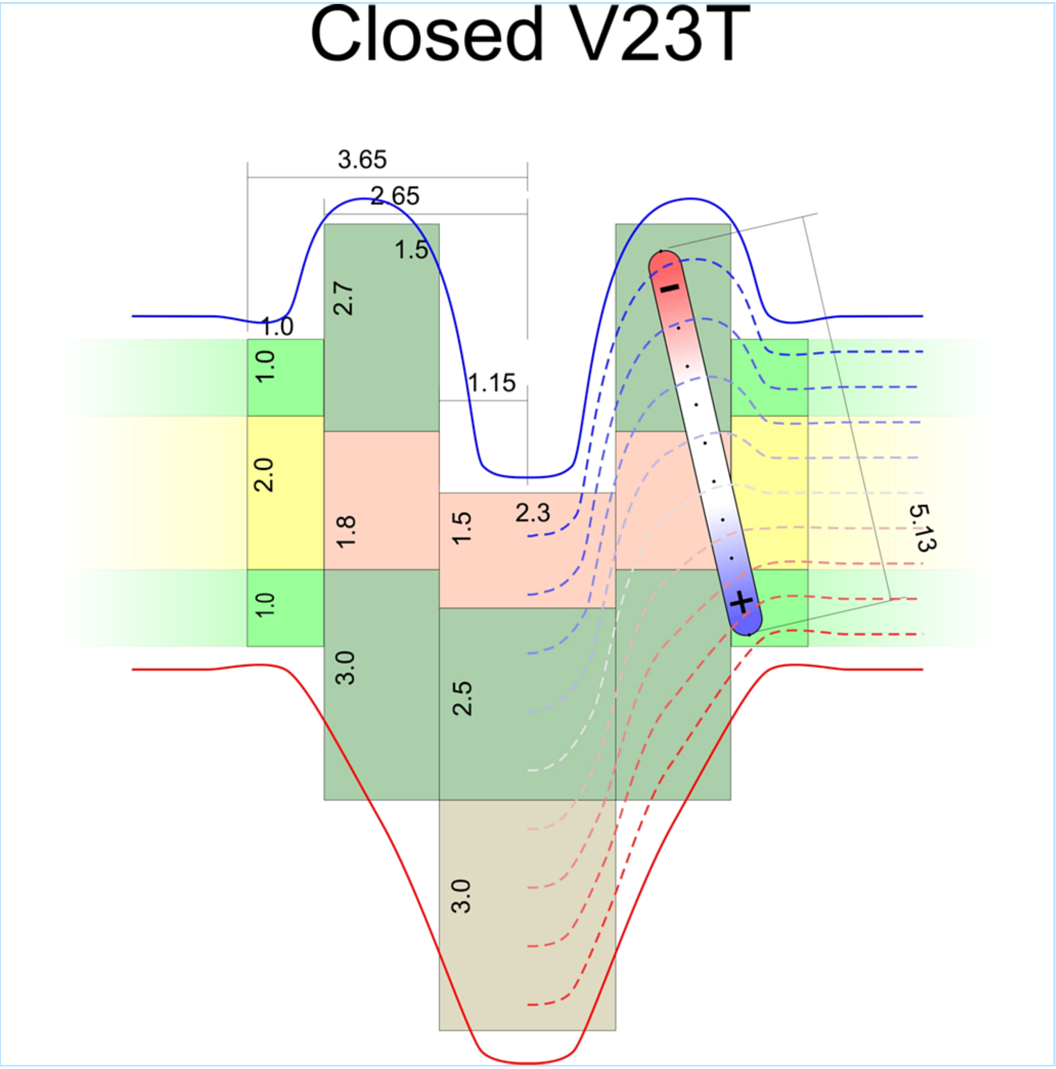

Geometry of the annular lipids ring

$r_a := 3.65\ nm$ External radius of the ring

$h_{ap} := 1.0\ nm$ Height of the polar region of the ring at the periplasmic side

$h_{an} := 2.0\ nm$ Height of the nonpolar region of the ring

$h_{ac} := 1.0\ nm$ Height of the polar region of the ring at the cytoplasmic side

Geometry of the protein barrel

$r_b := 2.65\ nm$ External radius of the protein barrel

$h_{bp} := 2.7\ nm$ Height of the polar region at the periplasmic side

$h_{bn} := 1.8\ nm$ Height of the nonpolar region of the barrel

$h_{bc} := 3.0\ nm$ Height of the polar region at the cytoplasmic side

Geometry of the ion-conucting pore

$r_i := 1.15\ \text{nm}$ Pore radius

$h_{in} := 1.5\ \text{nm}$ Height of the nonpolar gate region

$h_{ip} := 2.5\ \text{nm}$ Height of the polar region under the gate

$h_{ic} := 3.0\ \text{nm}$ Height of the C-terminal bundle

Geometry of the dipole in the external electric field

$p := 2675\ \text{D}$ Dipole moment of the channel

$d := 5.13\ \text{nm}$ Length of the channel dipole

$f_d := 1.0$ Fraction of the dipole moment in the external electric field

$f_{en} := 0.92$ Fraction of the electric field where the negative charge is

$f_{ep} := 0.15$ Fraction of the electric field where the positive charge is

Capacitors' energy

$$C_a := \frac{\varepsilon_0 \cdot \pi \cdot \left(r_a^2 - r_b^2\right)}{\frac{h_{ap}}{\varepsilon_{mp}} + \frac{h_{an}}{\varepsilon_{mn}} + \frac{h_{ac}}{\varepsilon_{mp}}} = 1.5924 \cdot 10^{-19}\ \text{F}$$

Capacitance of the annular lipid ring

$$Ec_a := -\frac{C_a \cdot U^2}{2} = -0.1557\ \text{k} \cdot T$$

Capacitive energy of the annular lipid ring

$$C_b := \frac{\varepsilon_0 \cdot \pi \cdot \left(r_b^2 - r_i^2\right)}{\frac{h_{bp}}{\varepsilon_{pp}} + \frac{h_{bn}}{\varepsilon_{pn}} + \frac{h_{bc}}{\varepsilon_{pp}}} = 3.5442 \cdot 10^{-19}\ \text{F}$$

Capacitance of the protein barrel

$$Ec_b := -\frac{C_b \cdot U^2}{2} = -0.3466\ \text{k} \cdot T$$

Capacitive energy of the protein barrel

$$C_i := \frac{\varepsilon_0 \cdot \pi \cdot r_i^2}{\frac{h_{in}}{\varepsilon_{pn}} + \frac{h_{ip}}{\varepsilon_{pp}} + \frac{h_{ic}}{\varepsilon_{pc}}} = 7.9196 \cdot 10^{-20}\ \text{F}$$

Capacitance of the ion-conducting pore

$$Ec_i := -\frac{C_i \cdot U^2}{2} = -0.0774\ \text{k} \cdot T$$

Capacitive energy of the ion-conducting pore

$$Ec := Ec_a + Ec_b + Ec_i = -0.5797\ \text{k} \cdot T$$

Capacitive energy of the whole system

$$Ec\_V23T_{closed} := Ec = -2.4011 \cdot 10^{-21}\ \text{J}$$

Same value assigned to the closed state

Dipole's energy

$q_d := \frac{p}{d} = 1.7393 \cdot 10^{-18}\ C$ $\quad q_d = 10.8561\ e$ Effective charge of the dipole

$Ed := f_d \cdot q_d \cdot U \cdot (f_{ep} - f_{en}) = -1.2054 \cdot 10^{-19}\ J$ $\quad Ed = -29.1014\ k \cdot T$ Energy of the dipole part exposed to the gradient of the potential

$Ed\_V23T_{closed} := Ed = -1.2054 \cdot 10^{-19}\ J$ Same value assigned to the closed state

---

Capacitive contribution for the Expanded conformation

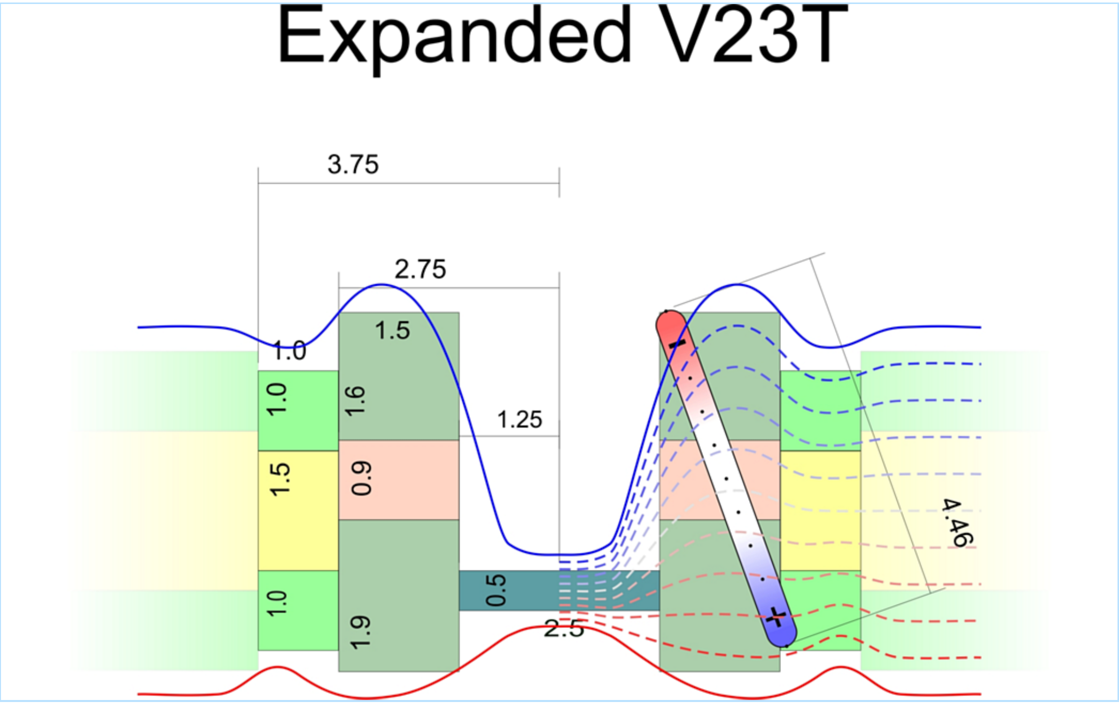

Geometry of the annular lipids ring

$r_a := 3.75$ nm External radius of the ring

$h_{ap} := 1.0$ nm Height of the polar region of the ring at the periplasmic side

$h_{an} := 1.5$ nm Height of the nonpolar region of the ring

$h_{ac} := 1.0$ nm Height of the polar region of the ring at the cytoplasmic side

Geometry of the protein barrel

$r_b := 2.75$ nm External radius of the protein barrel

$h_{bp} := 1.6$ nm Height of the polar region at the periplasmic side

$h_{bn} := 0.9$ nm Height of the nonpolar region of the barrel

$h_{bc} := 1.9$ nm Height of the polar region at the cytoplasmic side

Geometry of the ion-conucting pore

$r_i := 1.25$ nm Pore radius

$h_{ig} := 0.5$ nm Height of the hydrated polar gate region

Geometry of the dipole in the external electric field

$p := 2175$ D Dipole moment of the channel
$d := 4.46$ nm Length of the channel dipole
$f_d := 0.90$ Fraction of the dipole moment in the external electric field

$f_{en} := 1.0$ Fraction of the electric field where the negative charge is

$f_{ep} := 0.12$ Fraction of the electric field where the positive charge is

Capacitors' energy

Capacitors' energy

$$C_a := \frac{\varepsilon_0 \cdot \pi \cdot \left(r_a^{\ 2} - r_b^{\ 2}\right)}{\frac{h_{ap}}{\varepsilon_{mp}} + \frac{h_{an}}{\varepsilon_{mn}} + \frac{h_{ac}}{\varepsilon_{mp}}} = 2.1261 \cdot 10^{-19}\ F$$

Capacitance of the annular lipid ring

$$Ec_a := -\frac{C_a \cdot U^2}{2} = -0.2079\ k \cdot T$$

Capacitive energy of the annular lipid ring

$$C_b := \frac{\varepsilon_0 \cdot \pi \cdot \left(r_b^{\ 2} - r_i^{\ 2}\right)}{\frac{h_{bp}}{\varepsilon_{pp}} + \frac{h_{bn}}{\varepsilon_{pn}} + \frac{h_{bc}}{\varepsilon_{pp}}} = 6.8023 \cdot 10^{-19}\ F$$

Capacitance of the protein barrel

$$Ec_b := -\frac{C_b \cdot U^2}{2} = -0.6651\ k \cdot T$$

Capacitive energy of the protein barrel

$$C_i := \frac{\varepsilon_0 \cdot \pi \cdot r_i^{\ 2}}{\frac{h_{ig}}{\varepsilon_{pg}}} = 4.7787 \cdot 10^{-18}\ F$$

Capacitance of the ion-conducting pore

$$Ec_i := -\frac{C_i \cdot U^2}{2} = -4.6726\ k \cdot T$$

Capacitive energy of the ion-conducting pore

$$Ec := Ec_a + Ec_b + Ec_i = -5.5456\ k \cdot T$$

Capacitive energy of the whole system

$$Ec\_V23T_{expanded} := Ec = -2.297 \cdot 10^{-20}\ J$$

Same value assigned to the expanded state

Dipole's energy

$$q_d := \frac{p}{d} = 1.6267 \cdot 10^{-18}\ C$$

$q_d = 10.153\ e$ Effective charge of the dipole

$$Ed := f_d \cdot q_d \cdot U \cdot \left(f_{ep} - f_{en}\right) = -1.1595 \cdot 10^{-19}\ J$$

$Ed = -27.9941\ k \cdot T$ Energy of the dipole part exposed to the gradient of the potential

$$Ed\_V23T_{expanded} := Ed = -1.1595 \cdot 10^{-19}\ J$$

Same value assigned to the expanded state

Capacitive contribution for the Open conformation

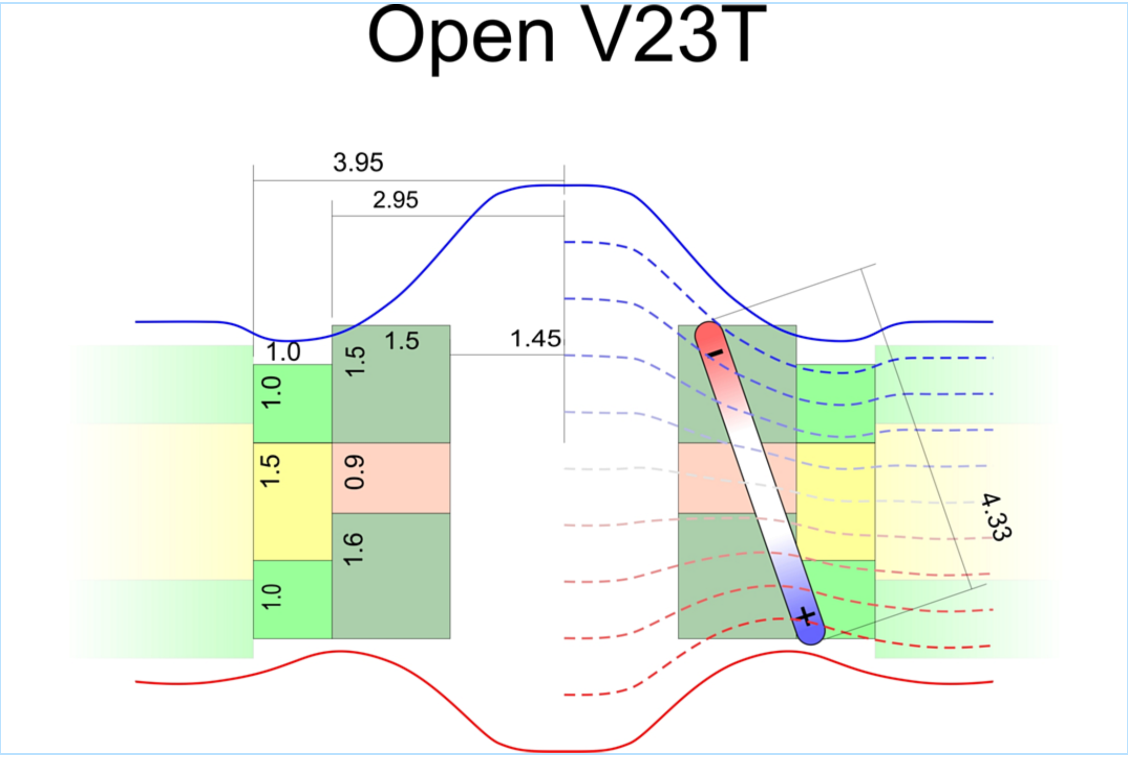

Geometry of the annular lipids ring

$r_a := 3.95$ nm External radius of the ring

$h_{ap} := 1.0$ nm Height of the polar region of the ring at the periplasmic side

$h_{an} := 1.5$ nm Height of the nonpolar region of the ring

$h_{ac} := 1.0$ nm Height of the polar region of the ring at the cytoplasmic side

Geometry of the protein barrel

$r_b := 2.95$ nm External radius of the protein barrel

$h_{bp} := 1.5$ nm Height of the polar region at the periplasmic side

$h_{bn} := 0.9$ nm Height of the nonpolar region of the barrel

$h_{bc} := 1.6$ nm Height of the polar region at the cytoplasmic side

Geometry of the ion-conucting pore

$r_i := 1.45$ nm Pore radius

Geometry of the dipole in the external electric field

$p := 2175$ D Dipole moment of the channel
$d := 4.33$ nm Length of the channel dipole
$f_d := 1.$ Fraction of the dipole moment in the external electric field

$f_{en} := 0.89$ Fraction of the electric field where the negative charge is

$f_{ep} := 0.03$ Fraction of the electric field where the positive charge is

Capacitors' energy

$$C_a := \frac{\varepsilon_0 \cdot \pi \cdot \left(r_a^{\,2} - r_b^{\,2}\right)}{\frac{h_{ap}}{\varepsilon_{mp}} + \frac{h_{an}}{\varepsilon_{mn}} + \frac{h_{ac}}{\varepsilon_{mp}}} = 2.257 \cdot 10^{-19}\ \mathrm{F}$$

Capacitance of the annular lipid ring

$$Ec_a := -\frac{C_a \cdot U^2}{2} = -0.2207\ \mathrm{k} \cdot T$$

Capacitive energy of the annular lipid ring

$$C_b := \frac{\varepsilon_0 \cdot \pi \cdot \left(r_b^{\,2} - r_i^{\,2}\right)}{\frac{h_{bp}}{\varepsilon_{pp}} + \frac{h_{bn}}{\varepsilon_{pn}} + \frac{h_{bc}}{\varepsilon_{pp}}} = 7.9127 \cdot 10^{-19}\ \mathrm{F}$$

Capacitance of the protein barrel

$$Ec_b := -\frac{C_b \cdot U^2}{2} = -0.7737\ \mathrm{k} \cdot T$$

Capacitive energy of the protein barrel

$$\frac{U_{pore}}{U} = \frac{R_{pore}}{R_{pore} + R_{access}} \qquad \frac{R_{pore}}{R_{pore} + R_{access}} = \frac{\frac{d}{\rho \cdot \pi \cdot r^2}}{\frac{d}{\rho \cdot \pi \cdot r^2} + \frac{2}{4 \cdot \rho \cdot r}} \qquad \frac{U_{pore}}{U} = \frac{1}{1 + \frac{r}{2 \cdot d}}$$

$$U_{pore} := \frac{U}{1 + \frac{r_i}{2 \cdot \left(h_{bp} + h_{bn} + h_{bc}\right)}} = 76.1905\ \mathrm{mV}$$

Voltage dropping on the pore,
based on Hall's equation

$$\frac{U_{pore}}{U} = 84.6561\ \%$$

Fraction of voltage dropping
on the pore

$$U_{access} := \frac{U - U_{pore}}{2} = 6.9048\ \mathrm{mV}$$

Voltage dropping on the access region at one side of the pore

$$C_i := \frac{\varepsilon_w \cdot \varepsilon_0 \cdot \pi \cdot r_i^{\,2}}{h_{bp} + h_{bn} + h_{bc}} = 1.1706 \cdot 10^{-18}\ \mathrm{F}$$

Capacitance of the ion-conducting pore

$$Ec_i := -\frac{C_i \cdot U_{pore}^{\,2}}{2} = -0.8203\ \mathrm{k} \cdot T$$

Capacitive energy of the ion-conducting pore

$$C_{i\_access} := 8 \cdot \varepsilon_w \cdot \varepsilon_0 \cdot r_i = 8.2231 \cdot 10^{-18}\ \mathrm{F}$$

Capacitance of one access region
of the ion-conducting pore

$$Ec_{i\_access} := -\frac{C_{i\_access} \cdot U_{access}^{\,2}}{2} = -0.0473\ \mathrm{k} \cdot T$$

Capacitive energy of one access region
of the ion-conducting pore

$$Ec := Ec_a + Ec_b + Ec_i + 2 \cdot Ec_{i\_access} = -1.9093\ \mathrm{k} \cdot T$$

Capacitive energy of the whole system

$$Ec\_V23T_{open} := Ec = -7.9084 \cdot 10^{-21}\ \mathrm{J}$$

Same value assigned to the open state

[illegible]

Dipole's_ energy

$q_d := \frac{p}{d} = 1.6755 \cdot 10^{-18}\ \mathrm{C}$ $\quad q_d = 10.4578\ \mathrm{e}$ Effective charge of the dipole

$Ed := f_d \cdot q_d \cdot U \cdot \left(f_{ep} - f_{en}\right) = -1.2969 \cdot 10^{-19}\ \mathrm{J}$ $\quad Ed = -31.3103\ \mathrm{k} \cdot T$ Energy of the dipole part exposed to the gradient of the potential

$Ed\_V23T_{open} := Ed = -1.2969 \cdot 10^{-19}\ \mathrm{J}$ Same value assigned to the open state

---

Summary for all the models

Capacitors' energy

WT

$Ec_{closed} = -2.0847 \cdot 10^{-21}\ J$

$Ec_{closed} = -0.5033\ k \cdot T$

$Ec_{open} = -7.6318 \cdot 10^{-21}\ J$

$Ec_{open} = -1.8426\ k \cdot T$

$\Delta Ec := Ec_{open} - Ec_{closed} = -5.5471 \cdot 10^{-21}\ J$

$\Delta Ec = -1.34\ k \cdot T$

$\Delta Ec_{experiment} := \left(-5.59 \cdot 10^{-21}\right)\ J = -1.3496\ k \cdot T$

$\Delta Ec_{experiment} = -1.35\ k \cdot T$

V23T

$Ec\_V23T_{closed} = -2.4011 \cdot 10^{-21}\ J$

$Ec\_V23T_{closed} = -0.5797\ k \cdot T$

$Ec\_V23T_{expanded} = -2.297 \cdot 10^{-20}\ J$

$Ec\_V23T_{expanded} = -5.5456\ k \cdot T$

$Ec\_V23T_{open} = -7.9084 \cdot 10^{-21}\ J$

$Ec\_V23T_{open} = -1.9093\ k \cdot T$

The energy for the time-averaged conductive state of V23T MscL is estimated as an average between the open and conductive states (as later is highly populated in V23T)

$$\Delta Ec\_V23T := \frac{Ec\_V23T_{open} + Ec\_V23T_{expanded}}{2} - Ec\_V23T_{closed} = -1.3038 \cdot 10^{-20}\ J$$

$\Delta Ec\_V23T = -3.15\ k \cdot T$

$\Delta Ec\_V23T_{experiment} := (-1.29) \cdot 10^{-20}\ J$

$\Delta Ec\_V23T_{experiment} = -3.11\ k \cdot T$

$$\frac{\Delta Ec\_V23T}{\Delta Ec} = 2.35$$

Relative energies (ratios) of the gating energies

$$\frac{\Delta Ec\_V23T_{experiment}}{\Delta Ec_{experiment}} = 2.31$$

$$\frac{\Delta Ec}{\Delta Ec_{experiment}} = 0.99$$

$$\frac{\Delta Ec_{experiment}}{\Delta Ec} = 1.01$$

$$\frac{\Delta Ec\_V23T}{\Delta Ec\_V23T_{experiment}} = 1.01$$

$$\frac{\Delta Ec\_V23T_{experiment}}{\Delta Ec\_V23T} = 0.99$$

Dipole's energy

WT

$Ed_{closed} = -1.2148 \cdot 10^{-19}\ J$

$Ed_{closed} = -29.3301\ k \cdot T$

$Ed_{open} = -1.2969 \cdot 10^{-19}\ J$

$Ed_{open} = -31.3103\ k \cdot T$

$\Delta Ed := Ed_{open} - Ed_{closed} = -8.2017 \cdot 10^{-21}\ J$

$\Delta Ed = -1.98\ k \cdot T$

$\Delta Ed_{experiment} := (-7.98) \cdot 10^{-21}\ J = -1.9266\ k \cdot T$

$\Delta Ed_{experiment} = -1.93\ k \cdot T$

V23T

$Ed\_V23T_{closed} = -1.2054 \cdot 10^{-19}\ J$

$Ed\_V23T_{closed} = -29.1014\ k \cdot T$

$Ed\_V23T_{expanded} = -1.1595 \cdot 10^{-19}\ J$

$Ed\_V23T_{expanded} = -27.9941\ k \cdot T$

$Ed\_V23T_{open} = -1.2969 \cdot 10^{-19}\ J$

$Ed\_V23T_{open} = -31.3103\ k \cdot T$

The energy for the time-averaged conductive state of V23T MscL is estimated as an average between the open and conductive states (as later is highly populated in V23T)

$$\Delta Ed\_V23T := \frac{Ed\_V23T_{open} + Ed\_V23T_{expanded}}{2} - Ed\_V23T_{closed} = -2.2813 \cdot 10^{-21}\ J$$

$\Delta Ed\_V23T = -0.55\ k \cdot T$

$\Delta Ed\_V23T_{experiment} := (-2.21) \cdot 10^{-21}\ J$

$\Delta Ed\_V23T_{experiment} = -0.53\ k \cdot T$

Relative energies (ratios) of the gating energies

$$\frac{\Delta Ed\_V23T}{\Delta Ed} = 0.28$$

$$\frac{\Delta Ed\_V23T_{experiment}}{\Delta Ed_{experiment}} = 0.28$$

$$\frac{\left(\frac{\Delta Ed\_V23T}{\Delta Ed}\right)}{\left(\frac{\Delta Ed\_V23T_{experiment}}{\Delta Ed_{experiment}}\right)} = 1.0043$$

$$\frac{\Delta Ed}{\Delta Ed_{experiment}} = 1.03$$

$$\frac{\Delta Ed_{experiment}}{\Delta Ed} = 0.97$$

$$\frac{\Delta Ed\_V23T}{\Delta Ed\_V23T_{experiment}} = 1.03$$

$$\frac{\Delta Ed\_V23T_{experiment}}{\Delta Ed\_V23T} = 0.97$$

Contributions of the dipole and capacitive components to the energy of MscL channel in the electric field at the negative potential

The purpose of these calculations is a qualitative estimate for the voltage-dependent energy contributions of different parts of the MscL-lipid system to the total energy cost of channel gating. While there are numerous simplifications to make the calculations straightforward, we believe the results grasp the essential trends in the electrostatic energy contributions during the gating transitions of MscL.

All the estimations were made in a continuum-dielectric simplification, with MscL channel and annular lipids represented as a set of concentric cylinders of a certain dielectric permeability (based on the typical values for polar and non-polar regions of proteins and lipid bilayer, see "Constants and material properties" section of this supplement), which streamlines the calculations of the capacitive energies.

The cylindrical geometry of the channel in the closed, expanded, and open states was roughly approximated from our published homology models for WT MscL and the models for V23T MscL developed using them as a template. For each model, the channel is represented as two concentric cylinders:
1) the inner cylinder represents the pore part.
For the closed state, it consists of the hydrophobic gate on the periplasmic side, hydrophilic region at the cytoplasmic side of the gate, and relatively hydrophobic cytoplasmic bundle formed by C-terminal residues.
For the expanded state (prominent only in V23T MscL), the pore cylinder is only a slim hydrophilic constriction formed by a hydrated polar residues. It prevents the passage of ions, but has high dielectric permeability.
For the fully open state, the inner cylinder is ion-conductive and completely filled by water.
2) the outside protein cylinder represents the transmembrane protein barrel.
It consists of three layers - polar periplasmic and cytoplasmic parts, with non-polar layer in between. This architecture is common for all the conformational states, however the cylinder decreases in height and increases in width as channel opens.
Lipid bilayer is modeled as two domains:
1) a cylinder of annular lipids surrounding the channel barrel. It has three layers - polar headgroup layers at the periplasmic and cytoplasmic sides and non-polar core of the lipid tails. The cylinders gets thinner on channels expansion and flattening.
2) a stable bulk membrane. It consists of the three layers as well (polar headgroups an non-polar core), however it remains stable through the whole conformational cycle, and therefore it was not included in calculations of the electrostatic energy changes.

The specific spatial dimensions for each model are specified in "Geometry of the annular lipids ring", "Geometry of the protein barrel", and "Geometry of the ion- conducting pore" subsections of the supplement, and visualized on the embedded images (all the distances are in nm).

The dipole moment of the transmembrane part of the channel was approximated as two charges located at the maximum and minimum of the electrostatic field calculated separately for each model in all-atom representation using PME (Particle Mesh Ewald) Electrostatics plugin of VMD, and overlaid on the cylindrical models. The values specific to each model can be found in "Geometry of the dipole in the external electric field" sections.

The applied electric field was assumed to have 90% drop of the external bulk values between the cytoplasmic and periplasmic surfaces of the protein or lipid (reflected in parameter "U" in the "Experimental Conditions" section. For the cylindrical representation, the equipotential levels at these surfaces were approximated by cubic splines smoothly connecting the points in the bulk at ~1 Angstrom distance from the centers of the protein or the lipid surfaces (see the images for visual representations). The intermediate potential levels were derived through linear interpolation between the potential boundaries and color -coded on the images from positive (blue) to negative (red).

For every model, the capacitive contribution to the electrostatic energy is detailed in it's "Capacitors' energy" section. The energy of each cylindrical part was estimate as an energy of a consecutive set of three cylindrical capacitors with the same area but different thickness and dielectric permeability (e.g. layers of polar headgroups and the non-polar core of the annular lipids cylinder). The contributions of all the concentric cylindrical parts (pore, barrel, and annular lipids) were added together for the net capacitive energy of a particular conformation.

The electrostatic contribution of the dipole of the protein barrel was then summed up with the capacitive contribution, taking into account the directionality of the field (i.e. the dipole part was favorable at the voltages with the positive potential at the periplasmic side, and unfavorable with the negative).

For all the models, the capacitive and dielectric components are integrated and compared to experimental data in the "Summary for all the models" section at the end of the supplement.

Constants and material properties

$\varepsilon_0 := 8.85 \cdot 10^{-12} \frac{F}{m}$	Dielectric permeability of vacuum
$\varepsilon_w := 80.1$	Relative permittivity of water
$\varepsilon_{mp} := 20$	Relative permittivity of the polar part of the membrane
$\varepsilon_{mn} := 2$	Relative permittivity of the nonpolar part of the membrane
$\varepsilon_{pp} := 30$	Relative permittivity of the polar part of the protein
$\varepsilon_{pn} := 7$	Relative permittivity of the nonpolar part of the protein
$\varepsilon_{pc} := 18$	Relative permittivity of the C-terminal bundle of the protein
$\varepsilon_{pg} := 55$	Relative permittivity of the expanded gate in V23T
$f_m := 0.9$	Fraction of the bulk membrane potential that drops "surface-to-surface" on protein and lipid.

Experimental conditions

$T := 300\ K$	Temperature
$\gamma := 10 \frac{dyne}{cm}$	Membrane tension
$U_e := (-100)\ mV$	Applied external transmembrane potential (pipette/periplasmic site)
$U := f_m \cdot U_e = -90\ mV$	Drop of the external transmembrane potential at the protein and lipid boundaries

Model parameters (WT MscL)

$Eo := 58\,k \cdot T = 2.4023 \cdot 10^{-19}\,J$ Open-closed energy difference

$\Delta A := 20\,nm^2$ Open-closed expansion area

Electrostatic contribution for the Closed conformation

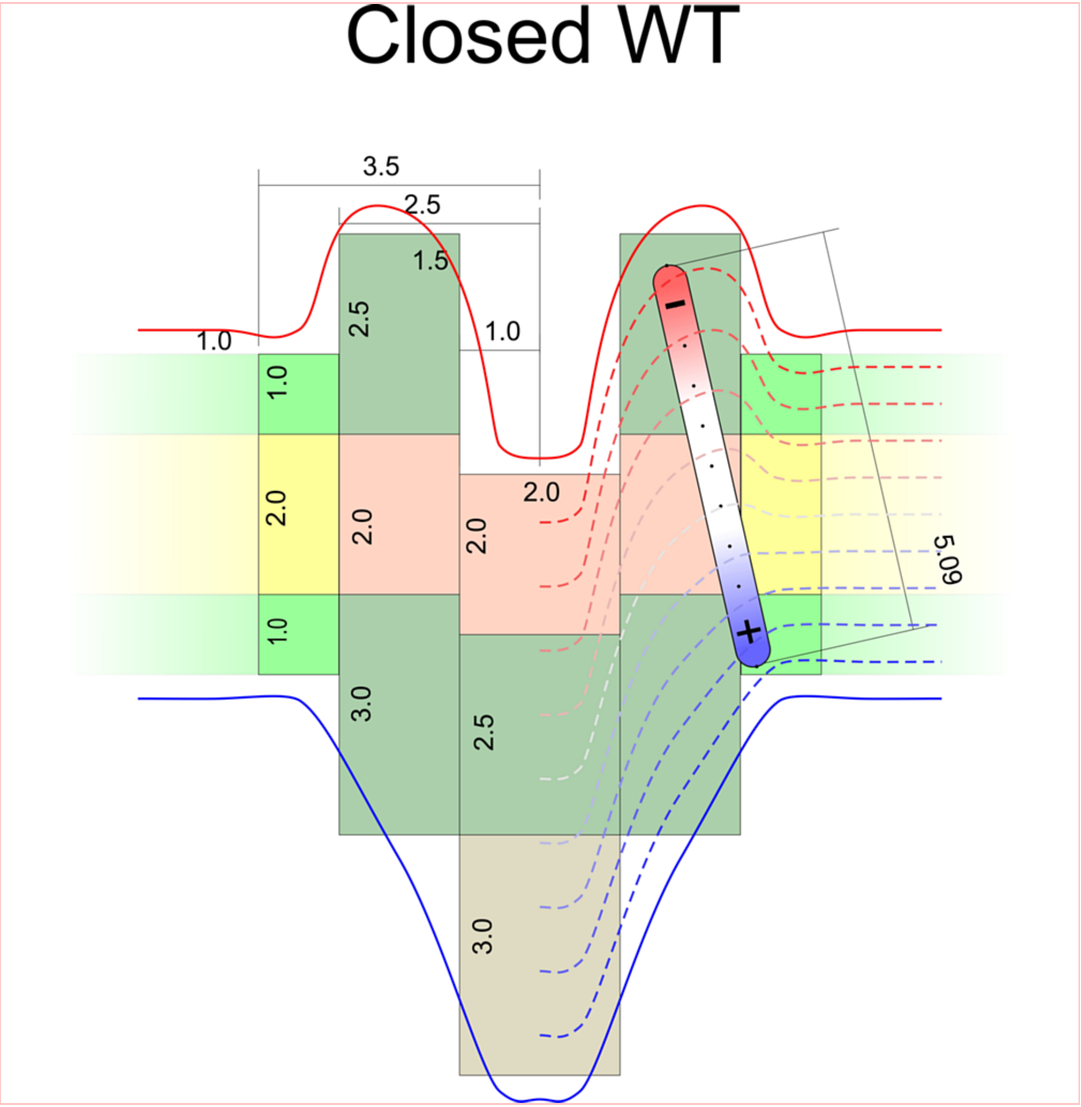

Geometry of the annular lipids ring

$r_a := 3.5\,nm$ External radius of the ring

$h_{ap} := 1.0\,nm$ Height of the polar region of the ring at the periplasmic side

$h_{an} := 2.0\,nm$ Height of the nonpolar region of the ring

$h_{ac} := 1.0\,nm$ Height of the polar region of the ring at the cytoplasmic side

Geometry of the protein barrel

$r_b := 2.5\,nm$ External radius of the protein barrel

$h_{bp} := 2.5\,nm$ Height of the polar region at the periplasmic side

$h_{bn} := 2.0\,nm$ Height of the nonpolar region of the barrel

$h_{bc} := 3.0\,nm$ Height of the polar region at the cytoplasmic side

Geometry of the ion-conducting pore

$r_i := 1.0\ \text{nm}$ Pore radius

$h_{in} := 2.0\ \text{nm}$ Height of the nonpolar gate region

$h_{ip} := 2.5\ \text{nm}$ Height of the polar region under the gate

$h_{ic} := 3.0\ \text{nm}$ Height of the C-terminal bundle

Geometry of the dipole in the external electric field

$p := 2675\ \text{D}$ Dipole moment of the channel

$d := 5.09\ \text{nm}$ Length of the channel dipole

$f_d := 1.0$ Fraction of the dipole moment in the external electric field

$f_{en} := 0.92$ Fraction of the electric field where the negative charge is

$f_{ep} := 0.15$ Fraction of the electric field where the positive charge is

Capacitors' energy

$$C_a := \frac{\varepsilon_0 \cdot \pi \cdot \left(r_a^{\,2} - r_b^{\,2}\right)}{\frac{h_{ap}}{\varepsilon_{mp}} + \frac{h_{an}}{\varepsilon_{mn}} + \frac{h_{ac}}{\varepsilon_{mp}}} = 1.5165 \cdot 10^{-19}\ \text{F}$$
Capacitance of the annular lipid ring

$$Ec_a := -\frac{C_a \cdot U^2}{2} = -0.1483\ \text{k} \cdot T$$
Capacitive energy of the annular lipid ring

$$C_b := \frac{\varepsilon_0 \cdot \pi \cdot \left(r_b^{\,2} - r_i^{\,2}\right)}{\frac{h_{bp}}{\varepsilon_{pp}} + \frac{h_{bn}}{\varepsilon_{pn}} + \frac{h_{bc}}{\varepsilon_{pp}}} = 3.112 \cdot 10^{-19}\ \text{F}$$
Capacitance of the protein barrel

$$Ec_b := -\frac{C_b \cdot U^2}{2} = -0.3043\ \text{k} \cdot T$$
Capacitive energy of the protein barrel

$$C_i := \frac{\varepsilon_0 \cdot \pi \cdot r_i^{\,2}}{\frac{h_{in}}{\varepsilon_{pn}} + \frac{h_{ip}}{\varepsilon_{pp}} + \frac{h_{ic}}{\varepsilon_{pc}}} = 5.1899 \cdot 10^{-20}\ \text{F}$$
Capacitance of the ion-conducting pore

$$Ec_i := -\frac{C_i \cdot U^2}{2} = -0.0507\ \text{k} \cdot T$$
Capacitive energy of the ion-conducting pore

$$Ec := Ec_a + Ec_b + Ec_i = -0.5033\ \text{k} \cdot T$$
Capacitive energy of the whole system

$Ec_{closed} := Ec = -2.0847 \cdot 10^{-21}\ J$ Same value assigned to the closed state

Dipole's energy

$q_d := \frac{p}{d} = 1.753 \cdot 10^{-18}\ C$ $q_d = 10.9415\ e$ Effective charge of the dipole

$Ed := f_d \cdot q_d \cdot U \cdot (f_{ep} - f_{en}) = 1.2148 \cdot 10^{-19}\ J$ $Ed = 29.3301\ k \cdot T$ Energy of the dipole part exposed to the gradient of the potential

$Ed_{closed} := Ed = 1.2148 \cdot 10^{-19}\ J$ Same value assigned to the closed state

Capacitive contribution for the Open conformation

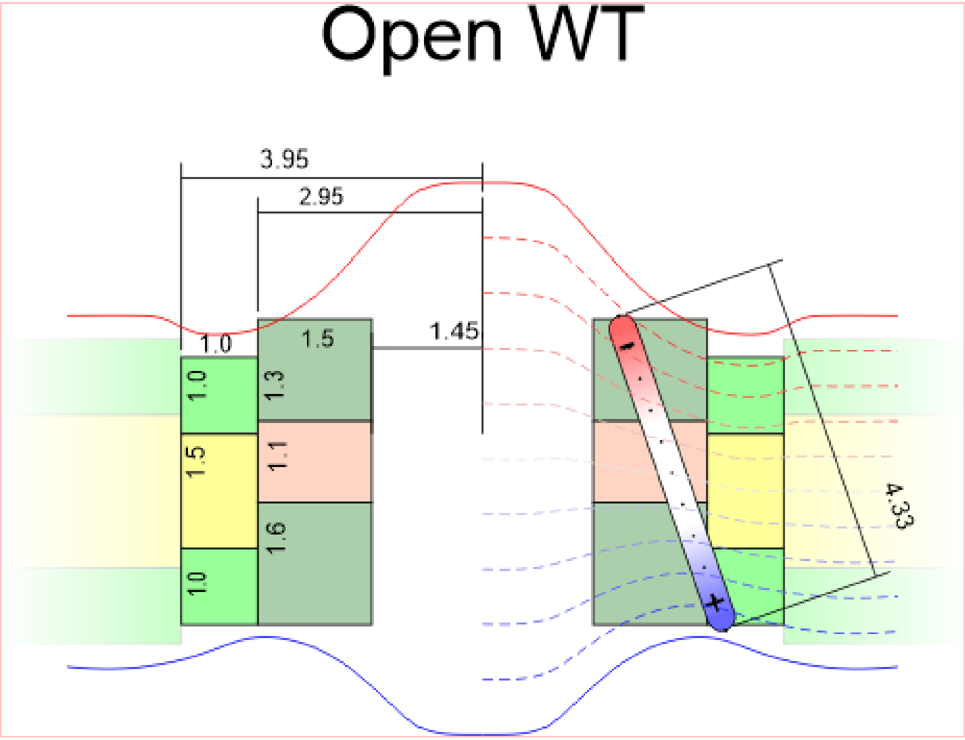

Geometry of the annular lipids ring

$r_a := 3.95$ nm External radius of the ring

$h_{ap} := 1.0$ nm Height of the polar region of the ring at the periplasmic side

$h_{an} := 1.5$ nm Height of the nonpolar region of the ring

$h_{ac} := 1.0$ nm Height of the polar region of the ring at the cytoplasmic side

Geometry of the protein barrel

$r_b := 2.95$ nm External radius of the protein barrel

$h_{bp} := 1.3$ nm Height of the polar region at the periplasmic side

$h_{bn} := 1.1$ nm Height of the nonpolar region of the barrel

$h_{bc} := 1.6$ nm Height of the polar region at the cytoplasmic side

Geometry of the ion-conducting pore

$r_i := 1.45$ nm Pore radius

Geometry of the dipole in the external electric field

$p := 2175$ D Dipole moment of the channel

$d := 4.33$ nm Length of the channel dipole

$f_d := 1.0$ Fraction of the dipole moment in the external electric field

$f_{en} := 0.89$ Fraction of the electric field where the negative charge is

$f_{ep} := 0.03$ Fraction of the electric field where the positive charge is

Capacitors' energy

$$C_a := \frac{\varepsilon_0 \cdot \pi \cdot \left(r_a^{\,2} - r_b^{\,2}\right)}{\frac{h_{ap}}{\varepsilon_{mp}} + \frac{h_{an}}{\varepsilon_{mn}} + \frac{h_{ac}}{\varepsilon_{mp}}} = 2.257 \cdot 10^{-19}\ \mathrm{F}$$

Capacitance of the annular lipid ring

$$Ec_a := -\frac{C_a \cdot U^2}{2} = -0.2207\ \mathrm{k} \cdot T$$

Capacitive energy of the annular lipid ring

$$C_b := \frac{\varepsilon_0 \cdot \pi \cdot \left(r_b^{\,2} - r_i^{\,2}\right)}{\frac{h_{bp}}{\varepsilon_{pp}} + \frac{h_{bn}}{\varepsilon_{pn}} + \frac{h_{bc}}{\varepsilon_{pp}}} = 7.2298 \cdot 10^{-19}\ \mathrm{F}$$

Capacitance of the protein barrel

$$Ec_b := -\frac{C_b \cdot U^2}{2} = -0.7069\ \mathrm{k} \cdot T$$

Capacitive energy of the protein barrel

$$\frac{U_{pore}}{U} = \frac{R_{pore}}{R_{pore} + R_{access}} \qquad \frac{R_{pore}}{R_{pore} + R_{access}} = \frac{\frac{d}{\rho \cdot \pi \cdot r^2}}{\frac{d}{\rho \cdot \pi \cdot r^2} + \frac{2}{4 \cdot \rho \cdot r}} \qquad \frac{U_{pore}}{U} = \frac{1}{1 + \frac{r}{2 \cdot d}}$$

$$U_{pore} := \frac{U}{1 + \frac{r_i}{2 \cdot \left(h_{bp} + h_{bn} + h_{bc}\right)}} = -76.1905\ \mathrm{mV}$$

Voltage dropping on the pore, based on Hall's equation

$$\frac{U_{pore}}{U} = 84.6561\ \%$$

Fraction of voltage dropping on the pore

$$U_{access} := \frac{U - U_{pore}}{2} = -6.9048\ \mathrm{mV}$$

Voltage dropping on the access region at one side of the pore

$$C_i := \frac{\varepsilon_w \cdot \varepsilon_0 \cdot \pi \cdot r_i^{\,2}}{h_{bp} + h_{bn} + h_{bc}} = 1.1706 \cdot 10^{-18}\ \mathrm{F}$$

Capacitance of the ion-conducting pore

$$Ec_i := -\frac{C_i \cdot U_{pore}^{\,2}}{2} = -0.8203\ \mathrm{k} \cdot T$$

Capacitive energy of the ion-conducting pore

$$C_{i\_access} := 8 \cdot \varepsilon_w \cdot \varepsilon_0 \cdot r_i = 8.2231 \cdot 10^{-18}\ \mathrm{F}$$

Capacitance of one access region of the ion-conducting pore

$$Ec_{i\_access} := -\frac{C_{i\_access} \cdot U_{access}^{\,2}}{2} = -0.0473\ \mathrm{k} \cdot T$$

Capacitive energy of one access region of the ion-conducting pore

$$Ec := Ec_a + Ec_b + Ec_i + 2 \cdot Ec_{i\_access} = -1.8426\ \mathrm{k} \cdot T$$

Capacitive energy of the whole system

$$Ec_{open} := Ec = -7.6318 \cdot 10^{-21}\ \mathrm{J}$$

Same value assigned to the open state

Dipole's energy

$q_d := \frac{p}{d} = 1.6755 \cdot 10^{-18}$ C $q_d = 10.4578$ e Effective charge of the dipole

$Ed := f_d \cdot q_d \cdot U \cdot (f_{ep} - f_{en}) = 1.2969 \cdot 10^{-19}$ J $Ed = 31.3103$ k·T Energy of the dipole part exposed to the gradient of the potential

$Ed_{open} := Ed = 1.2969 \cdot 10^{-19}$ J Same value assigned to the open state

---

Model parameters (V23T MscL)

$Eo := 41\,k \cdot T = 1.6982 \cdot 10^{-19}\,J$ Open-closed energy difference

$\Delta A := 18\,nm^2$ Open-closed expansion area

Capacitive contribution for the Closed conformation

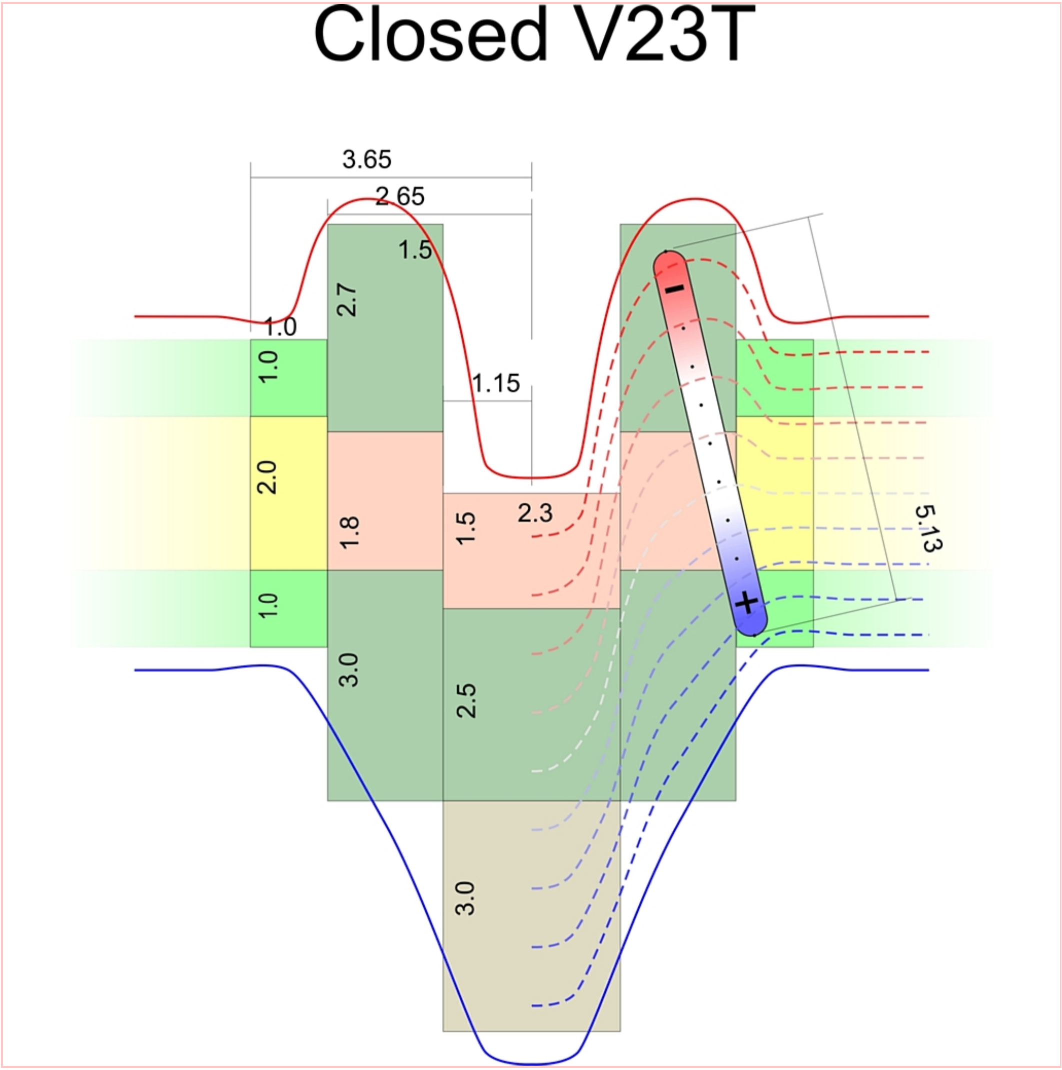

Geometry of the annular lipids ring

$r_a := 3.65\,nm$ External radius of the ring

$h_{ap} := 1.0\,nm$ Height of the polar region of the ring at the periplasmic side

$h_{an} := 2.0\,nm$ Height of the nonpolar region of the ring

$h_{ac} := 1.0\,nm$ Height of the polar region of the ring at the cytoplasmic side

Geometry of the protein barrel

$r_b := 2.65\,nm$ External radius of the protein barrel

$h_{bp} := 2.7\,nm$ Height of the polar region at the periplasmic side

$h_{bn} := 1.8\,nm$ Height of the nonpolar region of the barrel

$h_{bc} := 3.0\,nm$ Height of the polar region at the cytoplasmic side

Geometry of the ion-conducting pore

$r_i := 1.15 \text{ nm}$ Pore radius

$h_{in} := 1.5 \text{ nm}$ Height of the nonpolar gate region

$h_{ip} := 2.5 \text{ nm}$ Height of the polar region under the gate

$h_{ic} := 3.0 \text{ nm}$ Height of the C-terminal bundle

Geometry of the dipole in the external electric field

$p := 2675 \text{ D}$ Dipole moment of the channel

$d := 5.13 \text{ nm}$ Length of the channel dipole

$f_d := 1.0$ Fraction of the dipole moment in the external electric field

$f_{en} := 0.92$ Fraction of the electric field where the negative charge is

$f_{ep} := 0.15$ Fraction of the electric field where the positive charge is

Capacitors' energy

$$C_a := \frac{\varepsilon_0 \cdot \pi \cdot \left(r_a^{\,2} - r_b^{\,2}\right)}{\frac{h_{ap}}{\varepsilon_{mp}} + \frac{h_{an}}{\varepsilon_{mn}} + \frac{h_{ac}}{\varepsilon_{mp}}} = 1.5924 \cdot 10^{-19} \text{ F}$$

Capacitance of the annular lipid ring

$$Ec_a := -\frac{C_a \cdot U^2}{2} = -0.1557 \text{ k} \cdot T$$

Capacitive energy of the annular lipid ring

$$C_b := \frac{\varepsilon_0 \cdot \pi \cdot \left(r_b^{\,2} - r_i^{\,2}\right)}{\frac{h_{bp}}{\varepsilon_{pp}} + \frac{h_{bn}}{\varepsilon_{pn}} + \frac{h_{bc}}{\varepsilon_{pp}}} = 3.5442 \cdot 10^{-19} \text{ F}$$

Capacitance of the protein barrel

$$Ec_b := -\frac{C_b \cdot U^2}{2} = -0.3466 \text{ k} \cdot T$$

Capacitive energy of the protein barrel

$$C_i := \frac{\varepsilon_0 \cdot \pi \cdot r_i^{\,2}}{\frac{h_{in}}{\varepsilon_{pn}} + \frac{h_{ip}}{\varepsilon_{pp}} + \frac{h_{ic}}{\varepsilon_{pc}}} = 7.9196 \cdot 10^{-20} \text{ F}$$

Capacitance of the ion-conducting pore

$$Ec_i := -\frac{C_i \cdot U^2}{2} = -0.0774 \text{ k} \cdot T$$

Capacitive energy of the ion-conducting pore

$$Ec := Ec_a + Ec_b + Ec_i = -0.5797 \text{ k} \cdot T$$

Capacitive energy of the whole system

$$Ec\_V23T_{closed} := Ec = -2.4011 \cdot 10^{-21} \text{ J}$$

Same value assigned to the closed state

Dipole's_energy

$q_d := \frac{p}{d} = 1.7393 \cdot 10^{-18}\ C$ $q_d = 10.8561\ e$ Effective charge of the dipole

$Ed := f_d \cdot q_d \cdot U \cdot \left(f_{ep} - f_{en}\right) = 1.2054 \cdot 10^{-19}\ J$ $Ed = 29.1014\ k \cdot T$ Energy of the dipole part exposed to the gradient of the potential

$Ed\_V23T_{closed} := Ed = 1.2054 \cdot 10^{-19}\ J$ Same value assigned to the closed state

Capacitive contribution for the Expanded conformation

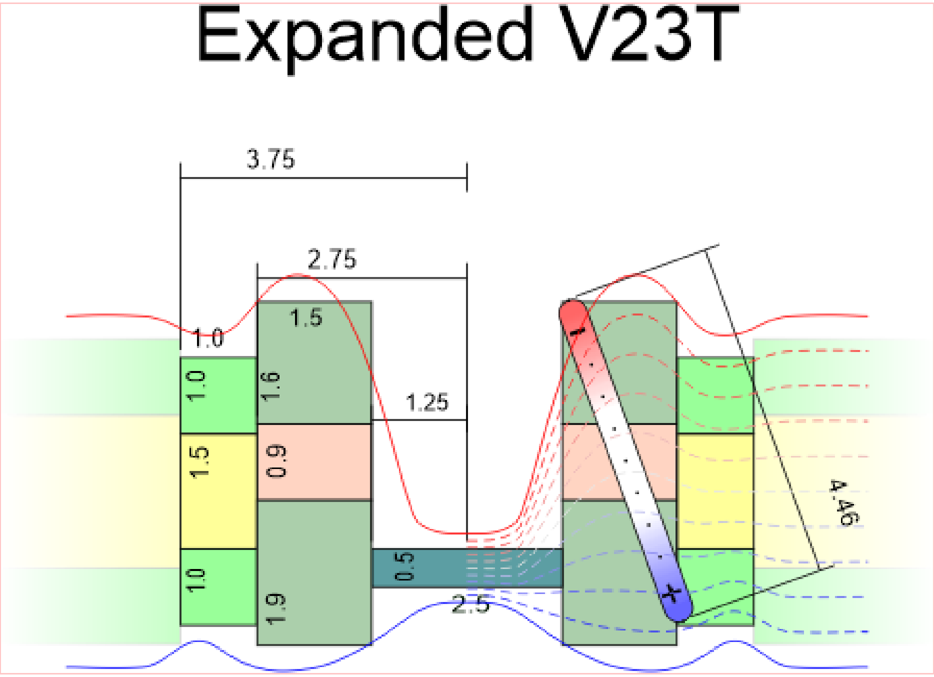

Geometry of the annular lipids ring

$r_a := 3.75$ nm External radius of the ring

$h_{ap} := 1.0$ nm Height of the polar region of the ring at the periplasmic side

$h_{an} := 1.5$ nm Height of the nonpolar region of the ring

$h_{ac} := 1.0$ nm Height of the polar region of the ring at the cytoplasmic side

Geometry of the protein barrel

$r_b := 2.75$ nm External radius of the protein barrel

$h_{bp} := 1.6$ nm Height of the polar region at the periplasmic side

$h_{bn} := 0.9$ nm Height of the nonpolar region of the barrel

$h_{bc} := 1.9$ nm Height of the polar region at the cytoplasmic side

Geometry of the ion-conducting pore

$r_i := 1.25$ nm Pore radius

$h_{ig} := 0.5$ nm Height of the hydrated polar gate region

Geometry of the dipole in the external electric field

$p := 2175$ D Dipole moment of the channel
$d := 4.46$ nm Length of the channel dipole
$f_d := 0.90$ Fraction of the dipole moment in the external electric field

$f_{en} := 1.0$ Fraction of the electric field where the negative charge is

$f_{ep} := 0.12$ Fraction of the electric field where the positive charge is

Capacitors' energy

Capacitors _ energy

$$C_a := \frac{\varepsilon_0 \cdot \pi \cdot \left(r_a^2 - r_b^2\right)}{\frac{h_{ap}}{\varepsilon_{mp}} + \frac{h_{an}}{\varepsilon_{mn}} + \frac{h_{ac}}{\varepsilon_{mp}}} = 2.1261 \cdot 10^{-19}\ \mathrm{F}$$

Capacitance of the annular lipid ring

$$Ec_a := -\frac{C_a \cdot U^2}{2} = -0.2079\ \mathrm{k} \cdot T$$

Capacitive energy of the annular lipid ring

$$C_b := \frac{\varepsilon_0 \cdot \pi \cdot \left(r_b^2 - r_i^2\right)}{\frac{h_{bp}}{\varepsilon_{pp}} + \frac{h_{bn}}{\varepsilon_{pn}} + \frac{h_{bc}}{\varepsilon_{pp}}} = 6.8023 \cdot 10^{-19}\ \mathrm{F}$$

Capacitance of the protein barrel

$$Ec_b := -\frac{C_b \cdot U^2}{2} = -0.6651\ \mathrm{k} \cdot T$$

Capacitive energy of the protein barrel

$$C_i := \frac{\varepsilon_0 \cdot \pi \cdot r_i^2}{\frac{h_{ig}}{\varepsilon_{pg}}} = 4.7787 \cdot 10^{-18}\ \mathrm{F}$$

Capacitance of the ion-conducting pore

$$Ec_i := -\frac{C_i \cdot U^2}{2} = -4.6726\ \mathrm{k} \cdot T$$

Capacitive energy of the ion-conducting pore

$$Ec := Ec_a + Ec_b + Ec_i = -5.5456\ \mathrm{k} \cdot T$$

Capacitive energy of the whole system

$$Ec\_V23T_{expanded} := Ec = -2.297 \cdot 10^{-20}\ \mathrm{J}$$

Same value assigned to the expanded state

Dipole's energy

$$q_d := \frac{p}{d} = 1.6267 \cdot 10^{-18}\ \mathrm{C} \qquad q_d = 10.153\ \mathrm{e}$$

Effective charge of the dipole

$$Ed := f_d \cdot q_d \cdot U \cdot \left(f_{ep} - f_{en}\right) = 1.1595 \cdot 10^{-19}\ \mathrm{J} \qquad Ed = 27.9941\ \mathrm{k} \cdot T$$

Energy of the dipole part exposed to the gradient of the potential

$$Ed\_V23T_{expanded} := Ed = 1.1595 \cdot 10^{-19}\ \mathrm{J}$$

Same value assigned to the expanded state

Capacitive contribution for the Open conformation

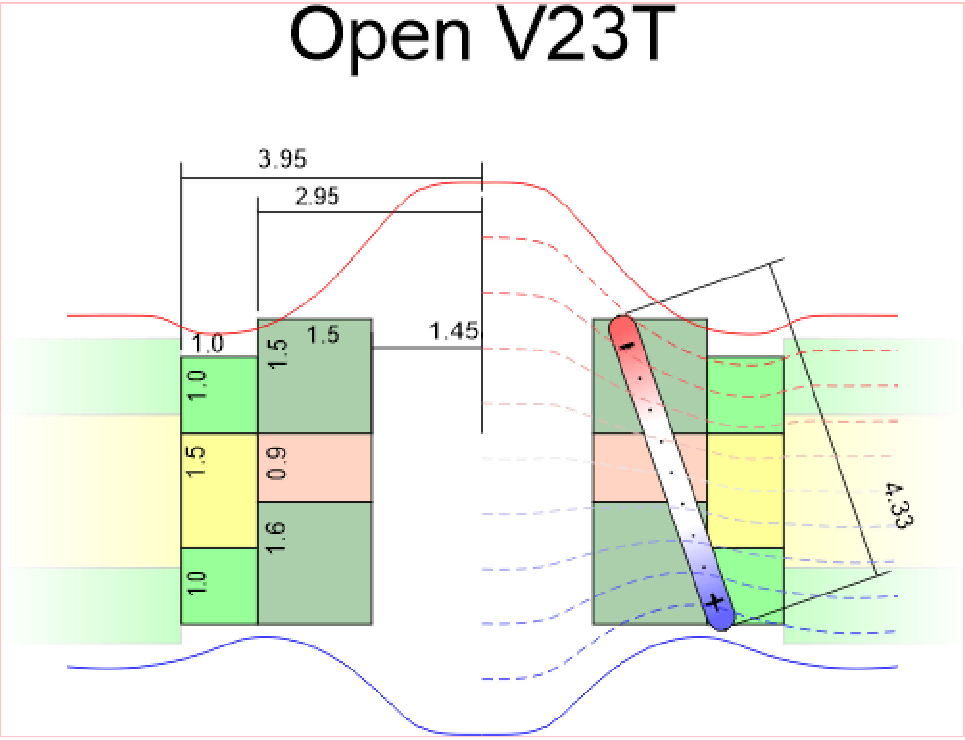

Geometry of the annular lipids ring

$r_a := 3.95\ nm$ External radius of the ring

$h_{ap} := 1.0\ nm$ Height of the polar region of the ring at the periplasmic side

$h_{an} := 1.5\ nm$ Height of the nonpolar region of the ring

$h_{ac} := 1.0\ nm$ Height of the polar region of the ring at the cytoplasmic side

Geometry of the protein barrel

$r_b := 2.95\ nm$ External radius of the protein barrel

$h_{bp} := 1.5\ nm$ Height of the polar region at the periplasmic side

$h_{bn} := 0.9\ nm$ Height of the nonpolar region of the barrel

$h_{bc} := 1.6\ nm$ Height of the polar region at the cytoplasmic side

Geometry of the ion-conducting pore

$r_i := 1.45\ nm$ Pore radius

Geometry of the dipole in the external electric field

$p := 2175\ D$ Dipole moment of the channel

$d := 4.33\ nm$ Length of the channel dipole

$f_d := 1.$ Fraction of the dipole moment in the external electric field

$f_{en} := 0.89$ Fraction of the electric field where the negative charge is

$f_{ep} := 0.03$ Fraction of the electric field where the positive charge is

Capacitors' energy

$$C_a := \frac{\varepsilon_0 \cdot \pi \cdot \left(r_a^{\,2} - r_b^{\,2}\right)}{\frac{h_{ap}}{\varepsilon_{mp}} + \frac{h_{an}}{\varepsilon_{mn}} + \frac{h_{ac}}{\varepsilon_{mp}}} = 2.257 \cdot 10^{-19}\ \mathrm{F}$$

Capacitance of the annular lipid ring

$$Ec_a := -\frac{C_a \cdot U^2}{2} = -0.2207\ \mathrm{k} \cdot T$$

Capacitive energy of the annular lipid ring

$$C_b := \frac{\varepsilon_0 \cdot \pi \cdot \left(r_b^{\,2} - r_i^{\,2}\right)}{\frac{h_{bp}}{\varepsilon_{pp}} + \frac{h_{bn}}{\varepsilon_{pn}} + \frac{h_{bc}}{\varepsilon_{pp}}} = 7.9127 \cdot 10^{-19}\ \mathrm{F}$$

Capacitance of the protein barrel

$$Ec_b := -\frac{C_b \cdot U^2}{2} = -0.7737\ \mathrm{k} \cdot T$$

Capacitive energy of the protein barrel

$$\frac{U_{pore}}{U} = \frac{R_{pore}}{R_{pore} + R_{access}} \qquad \frac{R_{pore}}{R_{pore} + R_{access}} = \frac{\frac{d}{\rho \cdot \pi \cdot r^2}}{\frac{d}{\rho \cdot \pi \cdot r^2} + \frac{2}{4 \cdot \rho \cdot r}} \qquad \frac{U_{pore}}{U} = \frac{1}{1 + \frac{r}{2 \cdot d}}$$

$$U_{pore} := \frac{U}{1 + \frac{r_i}{2 \cdot \left(h_{bp} + h_{bn} + h_{bc}\right)}} = -76.1905\ \mathrm{mV}$$

Voltage dropping on the pore, based on Hall's equation

$$\frac{U_{pore}}{U} = 84.6561\ \%$$

Fraction of voltage dropping on the pore

$$U_{access} := \frac{U - U_{pore}}{2} = -6.9048\ \mathrm{mV}$$

Voltage dropping on the access region at one side of the pore

$$C_i := \frac{\varepsilon_w \cdot \varepsilon_0 \cdot \pi \cdot r_i^{\,2}}{h_{bp} + h_{bn} + h_{bc}} = 1.1706 \cdot 10^{-18}\ \mathrm{F}$$

Capacitance of the ion-conducting pore

$$Ec_i := -\frac{C_i \cdot U_{pore}^{\,2}}{2} = -0.8203\ \mathrm{k} \cdot T$$

Capacitive energy of the ion-conducting pore

$$C_{i\_access} := 8 \cdot \varepsilon_w \cdot \varepsilon_0 \cdot r_i = 8.2231 \cdot 10^{-18}\ \mathrm{F}$$

Capacitance of one access region of the ion-conducting pore

$$Ec_{i\_access} := -\frac{C_{i\_access} \cdot U_{access}^{\,2}}{2} = -0.0473\ \mathrm{k} \cdot T$$

Capacitive energy of one access region of the ion-conducting pore

$$Ec := Ec_a + Ec_b + Ec_i + 2 \cdot Ec_{i\_access} = -1.9093\ \mathrm{k} \cdot T$$

Capacitive energy of the whole system

$$Ec\_V23T_{open} := Ec = -7.9084 \cdot 10^{-21}\ \mathrm{J}$$

Same value assigned to the open state

[illegible]

Dipole's_energy

$q_d := \frac{p}{d} = 1.6755 \cdot 10^{-18}\ \mathrm{C}$ $q_d = 10.4578\ \mathrm{e}$ Effective charge of the dipole

$Ed := f_d \cdot q_d \cdot U \cdot \left(f_{ep} - f_{en}\right) = 1.2969 \cdot 10^{-19}\ \mathrm{J}$ $Ed = 31.3103\ \mathrm{k} \cdot T$ Energy of the dipole part exposed to the gradient of the potential

$Ed\_V23T_{open} := Ed = 1.2969 \cdot 10^{-19}\ \mathrm{J}$ Same value assigned to the open state

---

Summary for all the models

Capacitors' energy

WT

$Ec_{closed} = -2.0847 \cdot 10^{-21}\ \mathrm{J}$

$Ec_{closed} = -0.5033\ \mathrm{k} \cdot T$

$Ec_{open} = -7.6318 \cdot 10^{-21}\ \mathrm{J}$

$Ec_{open} = -1.8426\ \mathrm{k} \cdot T$

$\Delta Ec := Ec_{open} - Ec_{closed} = -5.5471 \cdot 10^{-21}\ \mathrm{J}$

$\boxed{\Delta Ec = -1.34\ \mathrm{k} \cdot T}$

$\Delta Ec_{experiment} := \left(-5.59 \cdot 10^{-21}\right)\ \mathrm{J} = -1.3496\ \mathrm{k} \cdot T$

$\boxed{\Delta Ec_{experiment} = -1.35\ \mathrm{k} \cdot T}$

V23T

$Ec\_V23T_{closed} = -2.4011 \cdot 10^{-21}\ \mathrm{J}$

$Ec\_V23T_{closed} = -0.5797\ \mathrm{k} \cdot T$

$Ec\_V23T_{expanded} = -2.297 \cdot 10^{-20}\ \mathrm{J}$

$Ec\_V23T_{expanded} = -5.5456\ \mathrm{k} \cdot T$

$Ec\_V23T_{open} = -7.9084 \cdot 10^{-21}\ \mathrm{J}$

$Ec\_V23T_{open} = -1.9093\ \mathrm{k} \cdot T$

The energy for the time-averaged conductive state of V23T MscL is estimated as an average between the open and conductive states (as later is highly populated in V23T)

$\Delta Ec\_V23T := \dfrac{Ec\_V23T_{open} + Ec\_V23T_{expanded}}{2} - Ec\_V23T_{closed} = -1.3038 \cdot 10^{-20}\ \mathrm{J}$

$\boxed{\Delta Ec\_V23T = -3.15\ \mathrm{k} \cdot T}$

$\Delta Ec\_V23T_{experiment} := (-1.29) \cdot 10^{-20}\ \mathrm{J}$

$\boxed{\Delta Ec\_V23T_{experiment} = -3.11\ \mathrm{k} \cdot T}$

$\dfrac{\Delta Ec\_V23T}{\Delta Ec} = 2.35$

Relative energies (ratios) of the gating energies

$\dfrac{\Delta Ec\_V23T_{experiment}}{\Delta Ec_{experiment}} = 2.31$

$\dfrac{\Delta Ec}{\Delta Ec_{experiment}} = 0.99$

$\boxed{\dfrac{\Delta Ec_{experiment}}{\Delta Ec} = 1.01}$

$\dfrac{\Delta Ec\_V23T}{\Delta Ec\_V23T_{experiment}} = 1.01$

$\boxed{\dfrac{\Delta Ec\_V23T_{experiment}}{\Delta Ec\_V23T} = 0.99}$

Dipole's energy

WT

$Ed_{closed} = 1.2148 \cdot 10^{-19}\ J$

$Ed_{closed} = 29.3301\ k \cdot T$

$Ed_{open} = 1.2969 \cdot 10^{-19}\ J$

$Ed_{open} = 31.3103\ k \cdot T$

$\Delta Ed := Ed_{open} - Ed_{closed} = 8.2017 \cdot 10^{-21}\ J$

$\Delta Ed = 1.98\ k \cdot T$

$\Delta Ed_{experiment} := 7.98 \cdot 10^{-21}\ J = 1.9266\ k \cdot T$

$\Delta Ed_{experiment} = 1.93\ k \cdot T$

V23T

$Ed\_V23T_{closed} = 1.2054 \cdot 10^{-19}\ J$

$Ed\_V23T_{closed} = 29.1014\ k \cdot T$

$Ed\_V23T_{expanded} = 1.1595 \cdot 10^{-19}\ J$

$Ed\_V23T_{expanded} = 27.9941\ k \cdot T$

$Ed\_V23T_{open} = 1.2969 \cdot 10^{-19}\ J$

$Ed\_V23T_{open} = 31.3103\ k \cdot T$

The energy for the time-averaged conductive state of V23T MscL is estimated as an average between the open and conductive states (as later is highly populated in V23T)

$$\Delta Ed\_V23T := \frac{Ed\_V23T_{open} + Ed\_V23T_{expanded}}{2} - Ed\_V23T_{closed} = 2.2813 \cdot 10^{-21}\ J$$

$\Delta Ed\_V23T = 0.55\ k \cdot T$

$\Delta Ed\_V23T_{experiment} := 2.21 \cdot 10^{-21}\ J$

$\Delta Ed\_V23T_{experiment} = 0.53\ k \cdot T$

Relative energies (ratios) of the gating energies

$$\frac{\Delta Ed\_V23T}{\Delta Ed} = 0.28$$

$$\frac{\Delta Ed\_V23T_{experiment}}{\Delta Ed_{experiment}} = 0.28$$

$$\frac{\frac{\Delta Ed\_V23T}{\Delta Ed}}{\frac{\Delta Ed\_V23T_{experiment}}{\Delta Ed_{experiment}}} = 1.0043$$

$$\frac{\Delta Ed}{\Delta Ed_{experiment}} = 1.03$$

$$\frac{\Delta Ed_{experiment}}{\Delta Ed} = 0.97$$

$$\frac{\Delta Ed\_V23T}{\Delta Ed\_V23T_{experiment}} = 1.03$$

$$\frac{\Delta Ed\_V23T_{experiment}}{\Delta Ed\_V23T} = 0.97$$
